# Uncovering the liver’s role in immunity through RNA co-expression networks

**DOI:** 10.1007/s00335-016-9656-5

**Published:** 2016-07-11

**Authors:** Kylie K. Harrall, Katerina J. Kechris, Boris Tabakoff, Paula L. Hoffman, Lisa M. Hines, Hidekazu Tsukamoto, Michal Pravenec, Morton Printz, Laura M. Saba

**Affiliations:** 1 Department of Pharmaceutical Sciences, University of Colorado Skaggs School of Pharmacy and Pharmaceutical Sciences, Aurora, CO 80045 USA; 2 Department of Biostatistics and Informatics, University of Colorado School of Public Health, Aurora, CO 80045 USA; 3 Department of Pharmacology, University of Colorado School of Medicine, Aurora, CO 80045 USA; 4 Department of Biology, University of Colorado at Colorado Springs, Colorado Springs, CO 80918 USA; 5 Department of Pathology, Southern California Research Center for ALPD and Cirrhosis, Keck School of Medicine of USC, University of Southern California, Los Angeles, CA 90089 USA; 6 Department of Veterans Affairs, Great Los Angeles Healthcare System, Los Angeles, CA 90089 USA; 7 Department of Model Diseases, Institute of Physiology, Czech Academy of Sciences, Prague, Czech Republic; 8 Department of Pharmacology, University of California, San Diego, La Jolla CA, 92093 USA

## Abstract

Gene co-expression analysis has proven to be a powerful tool for ascertaining the organization of gene products into networks that are important for organ function. An organ, such as the liver, engages in a multitude of functions important for the survival of humans, rats, and other animals; these liver functions include energy metabolism, metabolism of xenobiotics, immune system function, and hormonal homeostasis. With the availability of organ-specific transcriptomes, we can now examine the role of RNA transcripts (both protein-coding and non-coding) in these functions. A systems genetic approach for identifying and characterizing liver gene networks within a recombinant inbred panel of rats was used to identify genetically regulated transcriptional networks (modules). For these modules, biological consensus was found between functional enrichment analysis and publicly available phenotypic quantitative trait loci (QTL). In particular, the biological function of two liver modules could be linked to immune response. The eigengene QTLs for these co-expression modules were located at genomic regions coincident with highly significant phenotypic QTLs; these phenotypes were related to rheumatoid arthritis, food preference, and basal corticosterone levels in rats. Our analysis illustrates that genetically and biologically driven RNA-based networks, such as the ones identified as part of this research, provide insight into the genetic influences on organ functions. These networks can pinpoint phenotypes that manifest through the interaction of many organs/tissues and can identify unannotated or under-annotated RNA transcripts that play a role in these phenotypes.

**Electronic supplementary material:**

The online version of this article (doi:10.1007/s00335-016-9656-5) contains supplementary material, which is available to authorized users.

## Introduction

With the emergence of the fields of precision medicine and systems genetics, the need for animal models and statistical methods to facilitate the translation of research from bench to bedside has grown (Malaney et al. 2014; Ritchie 2012). Identification of determinants of genetic susceptibility to disease and environmental toxins, and the integration of pharmacogenomic information are critical components of the NIH initiative to develop precision human medicine (Riley et al. 2015). However, genetic studies in humans are very costly in both time and resources; some of these costs can be attributed to the requirement of large sample sizes and the lack of a priori control over genetic and environmental variables. In addition, many human studies cannot be performed for ethical reasons. Rodent models not only provide opportunities to control and manipulate genetics and environmental factors, but they also provide the opportunity to investigate biological processes that cannot ethically be studied in humans. Rats are a prominent model for studying the mechanisms of disease, the impact of environmental factors, and new drug technologies (Aitman et al. 2008). Genetic models, rapid advances in sequencing technologies, and recent gene-editing techniques have allowed rats to remain a major resource for functional and mechanistic studies in medicine (Parker et al. 2014).

Historically, investigators have often used a single inbred strain or rodents from an outbred stock to examine biological mechanisms of disease (Festing 2014). Another approach, which has distinct advantages for systems genetic studies, is to use a panel of inbred or recombinant inbred (RI) rat strains (Printz et al. 2003). In such a panel, the varied, but known, genetic composition of the panel is relatively static and retained over generations. This ‘reproducibility’ of genetic background allows for the identification of interactions among multiple phenotypes, and their relationships to genetic variation. The accumulation of multiple behavioral, physiological, and molecular phenotypes over generations and across laboratories is essential for truly integrative systems genetics research.

One type of molecular phenotype that is of particular interest in systems genetics research is RNA transcript abundance. By quantitatively measuring RNA expression levels across an inbred rat panel, one can attempt to describe a functional relationship between genes through co-expression. The general theory of co-expression studies is that if the expression levels of two transcripts react to different genetic backgrounds in a similar manner, then the two transcripts are likely involved in a similar biological process (Allocco et al. 2004). One can use graph theory to describe the relationships among genes based on co-expression across different genetic backgrounds (such as in RI panels). For example, these co-expression networks can be modeled as robust scale-free gene networks (Ravasz et al. 2002; Weiss et al. 2012). Transcripts within a scale-free network are not connected at random, rather these networks are composed of many nodes with few connections, and a few hub nodes, which are connected to many other transcripts.

The crux of our work is based on the proposition that if a genetic locus influences both a behavioral/physiological trait and RNA expression levels in an identified transcriptional network, then the transcriptional network is likely to influence the behavioral/physiological trait (Saba et al. 2015). In the current work, we sought to answer one question: can one use information about the genetic locus of expression variation in a module (eigengene QTL), from a newly described co-expression network, to search phenotypic quantitative trait loci (QTL) databases for overlapping behavioral/physiologic QTLs and arrive at an informative relationship between a physiological/behavioral phenotype and the transcriptome.

**Fig. 1:**
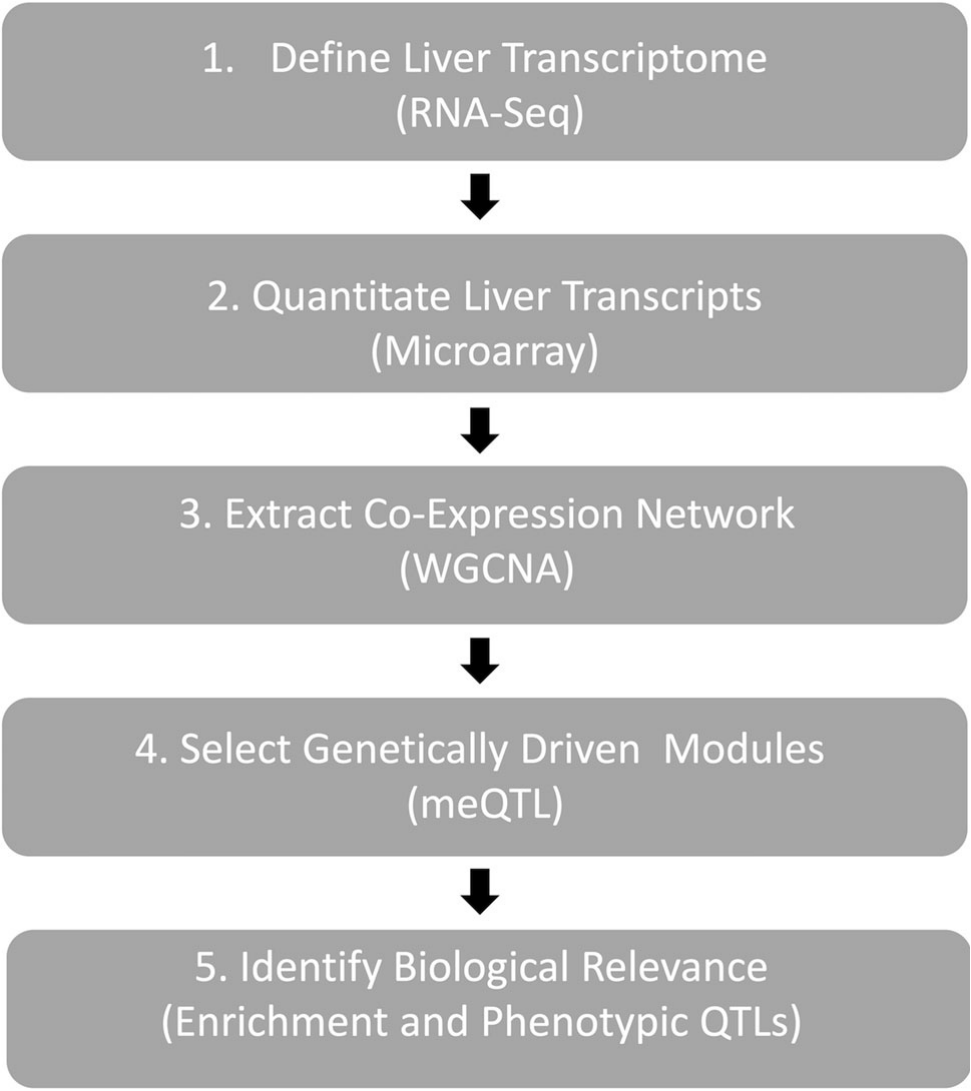
An outline of systems genetics approach. This workflow was used to identify genetically driven and biologically relevant liver modules

We utilized RI rats and quantitative systems genetics methods (network analysis of RNA expression levels) to group both well-annotated, under-annotated, and unannotated RNA transcripts into biologically relevant networks, and to link these networks to phenotypic traits through shared genetic influences (i.e., overlapping phenotypic and expression QTL). We focused on the liver transcriptome of the HXB/BXH RI rat panel. Our approach, outlined in Fig. 1, contained five steps: (1) define the liver transcriptome from the progenitor strains of the HXB/BXH RI rat panel (SHR and BN-Lx), (2) measure expression levels of the liver transcriptome in the HXB/BXH RI rat panel, (3) identify co-expression modules within the liver, (4) identify modules exhibiting high levels of genetic control, and (5) identify the biological relevance of genetically driven modules.

This approach led to the discovery of two genetically driven co-expression liver modules that were associated with the immune system. Although the liver is often associated with metabolism, it has many roles in the body (Higuchi and Gores 2003). This research has uncovered a genetic pathway associated with the lesser known function of liver, innate immunity (Gao et al. 2008; Racanelli and Rehermann 2006), and demonstrates how system genetics approaches, such as the one discussed in this manuscript, have the potential to aid in the discovery of genetic influences on biological functions and to expand current research directions.

## Materials and methods

### Animals

RI panels are produced by first crossing two genetically and phenotypically diverse inbred strains to create an F1 generation with one chromosome copy from each parent. At the F2 generation, as a result of recombinant events, each animal’s DNA sequence becomes a mosaic of the original DNA sequences of the two parental strains. After many generations of brother/sister mating, an RI panel is produced that contains a large number of inbred strains. All rats within a strain are genetically identical (similar to monozygotic twins), and that genetic identity is retained over generations. Rats from different strains within the panel share approximately 50 % of the genetic sequence of the parental strains, much like dizygotic twins or siblings (Printz et al. 2003).

In this study, we assessed rats from the HXB/BXH RI panel, which was originally developed in the Czech Republic by Vladimir Kren (Institute of Biology of Charles University) and Michal Pravenec (Institute of Physiology of the Czech Academy of Sciences). This panel was generated using gender reciprocal crossing between the congenic Brown Norway strain with polydactyly-luxate syndrome (BN-Lx/Cub) and the spontaneous hypertensive rat strain (SHR/OlaIpcv), with sixty generations of brother/sister mating after the F2 generation (Printz et al. 2003). Although this panel was originally developed to examine differences in cardiovascular traits, many other traits vary across the strains and have been studied extensively (Bielavská et al. 2002, 2002; Conti et al. 2004; Kunes et al. 1994; Pravenec et al. 2004; Vanderlinden et al. 2014).

### Identification of RNA transcripts in the liver

#### Whole liver RNA sequencing

Male rats from substrains of the original parental strains of the HXB/BXH RI panel, BN-Lx/CubPrin (BN-Lx) and SHR/OlaIpcvPrin (SHR) were used for RNA-Seq analyses. Rats were maintained and bred at the University of California, San Diego. Three male rats from each parental strain (70–90 days old) were quickly anesthetized with isofluorane/air and decapitated according to a protocol approved by the UCSD IACUC. Livers were quickly frozen in liquid nitrogen, and they were shipped to the University of Colorado for RNA extraction and library preparation.

For each of the six liver samples, total RNA was extracted with the RNeasy Midi Kit; additional cleanup was performed using the RNeasy Mini Kit (Qiagen, Valencia, CA, USA). External RNA Controls Consortium (ERCC) Synthetic Spike-Ins (ThermoFisher Scientific, Wilmington, DE, USA) were added to the extracted RNA; 4 µl was added from a 1:100 dilution of either Mix 1 or 2. Sequencing libraries were constructed using the Illumina TruSeq Stranded RNA Sample Preparation kit (Illumina, San Diego, CA, USA), in accordance with the manufacturer’s protocol, and library quality was assessed using the Agilent Bioanalyzer 2100 (Agilent Technologies, Santa Clara, CA, USA). Ribosomal RNA depletion was carried out as part of the Illumina TruSeq Stranded Total RNA Library Prep Kit, which includes the Ribo-Zero ribosomal RNA reduction chemistry. Samples were sequenced (2x100 paired-end reads) on the Illumina HiSeq2000 (Illumina, San Diego, CA, USA) with two samples multiplexed per lane.

For each of the livers of the rats from the BN-Lx and SHR parental strains, the raw reads were trimmed for quality and adaptors using trim-galore (http://www.bioinformatics.babraham.ac.uk/projects/trim_galore/, version 0.4.0). The trimmed reads were aligned using Tophat2 (Kim et al. 2013) (Version 2.1.0) with default parameters. The reads were initially aligned to rRNA from the RepeatMasker database (Smit et al. 1996), which was downloaded from the UCSC Genome Browser (Karolchik et al. 2004); all of the paired-end reads with either end aligned to those sequences were eliminated from the analysis. If reads did not align to rRNA, they were aligned to the rn5 version of their respective strain-specific genomes. The strain-specific genomes were constructed with DNA-Seq data from male brains of the BN-Lx/CubPrin and SHR/OlaIpcvPrin strains (Hermsen et al. 2015), and they are publicly available on the PhenoGen website [http://phenogen.ucdenver.edu (Saba et al. 2015)].

#### Transcriptome reconstruction in progenitor strains

For reconstructing the rat liver transcriptome, reads from all biological replicates within an inbred strain were combined. Strain-specific transcriptomes were then reconstructed using the Cufflinks algorithm (Trapnell et al. 2012) (Version 2.2.1), with the rn5 Ensembl Rat Transcriptome (Roberts et al. 2011; Trapnell et al. 2010) as a guide. Mitochondrial chromosomes were initially masked due to the depth of reads within this small chromosome, which would dramatically increase computational burden.

Within the strain-specific transcriptomes, “high-confidence transcripts” (defined below) were identified using estimated expression levels and transcript length. Fragments per kilobase of transcript per million mapped reads (FPKM) were compared across transcripts, and a threshold was set to filter out low confidence transcripts. FPKM values were calculated from the combined reads within a strain. The focus was to qualitatively describe the transcriptome without the restriction that the transcript be expressed in multiple animals. Along with the FPKM threshold, transcripts had to exceed a transcribed length of 300 base pairs (bp); this was the length required to survive size selection during RNeasy RNA extraction and the gel-size selection step within the Illumina TruSeq protocol.

For mapping and annotation, the resulting high-confidence BN-Lx and SHR transcriptomes were merged to create a single combined transcriptome. Ensembl-annotated mitochondrial transcripts were reintroduced at this stage, so that these transcripts were included in the final mapped transcriptome. Overlapping transcripts between the two strain-specific transcriptomes were identified and merged into a single transcript if they occurred on the same strand (or one strand was not designated), and they met at least one of the following requirements: (1) all of the splice junctions matched, or (2) both transcripts contained only one exon, and when the two transcripts were compared, their transcription start and stop sites were within 100 bp of each other. After merging transcripts that overlapped, transcripts (i.e., splice variants) were grouped into genes. We determined that two transcripts were from the same gene if their transcription start sites matched exactly, their transcription stop sites matched exactly, or at least one splice junction matched exactly.

Because the Cufflinks algorithm accommodates reads aligned to multiple transcripts by splitting their read count among the different transcripts (often multiple splice variants of the same gene) in a probabilistic manner, the addition of an alternative transcript to a transcriptome may alter the expression estimate of the original transcript(s). To identify the most robust set of transcripts, the combined transcriptome of high-confidence transcripts was quantitated separately in each strain. These transcripts were quantitated using coverage, i.e., the average number of reads per nucleotide across the whole transcript. If a transcript’s coverage dropped below an average of 50 reads per nucleotide in each of the strains when considering all transcripts in the combined transcriptome, it was dropped from the combined transcriptome. This iteration of quantitation and reduction of the transcriptome was repeated until less than 5 % of the remaining transcripts fell below our coverage threshold.

### Quantitation of RNA expression in the livers of the HXB/BXH recombinant inbred panel

#### Microarray expression measurements

Male rats from 21 strains of the HXB/BXH RI panel were used for exon array analyses. Like the parental rat strains used for RNA-Seq, rats from the RI panel were maintained and bred at the University of California, San Diego (UCSD). Three to four male rats from each strain (70–90 days old) were quickly anesthetized with isofluorane/air and decapitated according to a protocol approved by the UCSD IACUC. Livers were quickly extracted and frozen on dry ice or in liquid nitrogen. Livers were shipped to the University of Colorado for RNA extraction and microarray processing.

RNA was extracted from each whole liver using the RNeasy Midi Kit (Qiagen) and the RNeasy Mini kit (Qiagen) for cleanup. cDNA from the liver of each individual rat was hybridized to a separate Affymetrix Rat Exon 1.0 ST array following the manufacturer’s protocol. All processed array data were examined for quality using the tools outlined in detail on the PhenoGen website.

Probe sequences for the Affymetrix Rat Exon 1.0 ST Array were obtained from the Affymetrix website (http://www.affymetrix.com/). High-integrity probes were identified based on their alignment to the genome and known genomic variants in the BN-Lx and SHR strains (Saba et al. 2015). For example, probes were removed from the analysis if they did not align perfectly and singularly to the rn5 rat genome. Probes were also removed if they aligned to a region that harbored an SNP, small insert, or small deletion between the BN-Lx or SHR strains, when compared to the Brown Norway (BN) reference genome (Hermsen et al. 2015). Entire probe sets were eliminated if less than three probes remained after filtering. This array mask is publicly available on the PhenoGen website.

The full set of probe sets on the Affymetrix array targets many different regions of the genome; this include annotated genes, and unannotated regions with sequence characteristics that predict possible transcription. This breadth of coverage allows for the quantitation of both annotated and unannotated transcripts in the reconstruction. To do this, probe sets from the exon array were mapped to transcripts in the reconstructed liver transcriptome. Probe sets were retained if their targeted region was exclusively and entirely within a gene. This allowed for the selection of probe sets specific for genes that are expressed in the liver of naïve BN-Lx and SHR rats, including probe sets that targeted unannotated genes.

All of the probes that targeted a particular gene were grouped into a single expression estimate, which is called a gene cluster throughout this manuscript. The mapping of probe sets to gene clusters is available on the PhenoGen website. Genes with at least one “high-confidence” transcript in the reconstruction, which precisely matched a transcript annotated in the rn5 Ensembl Rat Transcriptome, were associated with gene information for that annotated transcript. Affymetrix Power Tools (http://www.affymetrix.com/) was used to implement an RMA-sketch algorithm to estimate expression levels for individual gene clusters. Correction for batch effects was executed using the ComBat algorithm (Johnson et al. 2007). We required that at least 5 % of samples had expression above background (the detection above background (DABG) *p* value <0.0001) for a gene cluster to be retained for further analyses.

### Network analysis

#### Weighted gene correlation network analysis (WGCNA)

Strain means of gene clusters were used for co-expression analysis. Each gene cluster represents a singular gene product, which is defined as all of the RNA transcripts derived from a particular gene. An unsigned weighted gene correlation network was constructed using the R package WGCNA (Langfelder and Horvath 2008) (version 1.49). A co-expression similarity matrix was derived by calculating pairwise Pearson correlation coefficients between the expression levels of genes across strains. This similarity matrix was subsequently transformed into a scale-free connectivity network by raising each correlation coefficient to a power of 7; this threshold was chosen using criteria and methods from Langfelder, Horvath, and Zhang (Langfelder and Horvath 2008; Zhang and Horvath 2005) for modeling a scale-free network. In an effort to avoid spurious connections within our networks, a topological overlap map (TOM) was used to assess both the direct connection between two gene products and the indirect connection between the two gene products based on the similarity of their relationships with other gene products (Langfelder 2013). The minimum module size was set at 5 to allow for smaller modules, compared to the default setting of 30; subsequently, modules were merged if their eigengenes were highly correlated using a threshold of 0.75. Gene product co-expression modules were identified with dynamic tree cutting (Langfelder et al. 2008); this method identifies large clusters of gene products with an initial static cut, then refines the large clusters by recursively splitting them into subclusters. Principal Components Analysis was used to calculate an eigengene (the first principal component) for each co-expression module. Each eigengene represents the expression pattern across strains for the module, and the accuracy of these representations can be quantified as the proportion of variance of gene expression across strains explained by the eigengene.

Within each module, we calculated the intramodular connectivity and the number of connections for each gene product (Zhang and Horvath 2005). Intramodular connectivity was defined as the sum of connection strengths with gene products within a module associated with a gene product of interest, and the gene product with highest connectivity within each module was identified as the hub gene product. The number of intramodular connections of a gene product represents the number of gene products within the module that are correlated with that gene product (correlation coefficient > |0.5|).

### Selection of genetically driven modules

To identify genetically driven modules in the liver, modules were required to have: (1) an eigengene that explained over 50 % of module variance (Langfelder et al. 2011), and (2) a significant module eigengene quantitative trait loci (meQTL), i.e., genome-wide *p* value <0.05.

meQTLs were identified by testing for an association between a module eigengene and a set of genomic markers (i.e., strain distribution patterns) for the HXB/BXH panel. This set of markers was downloaded from PhenoGen, and further details are available in the study by Saba et al. (2015). Associations were calculated with R/qtl (Broman et al. 2003) (Version 1.28–4) using marker regression with a genome-wide LOD significance threshold based on 1000 permutations (Churchill and Doerge 1994).

### Identifying biological relevance of modules

We used two independent public databases to uncover the biological function of each genetically driven module. First, we used PANTHER (http://pantherdb.org/) to find significant enrichment for pathways or gene ontology (GO) terms among the annotated gene products of the module (Mi et al. 2016). If a module was significantly enriched for a GO term or PANTHER pathway, we used the Rat Genome Database’s (RGD) QTL search (Shimoyama et al. 2015) (http://rgd.mcw.edu/) to find phenotypic QTLs in the same genomic location as the meQTL.

#### PANTHER gene ontology and enrichment

PANTHER’s statistical overrepresentation test (Mi et al. 2013) was used to identify enrichment (adjusted *p* < 0.05) of PANTHER pathways and GO terms, which were categorized as either related to biological processes, molecular functions, or cellular components. Because this type of analysis increases in power as more gene products are included, we required that each module contain at least ten Ensembl annotated gene products. This type of analysis identifies terms or pathways that are statistically overrepresented by gene products represented within a module. PANTHER uses the binomial distribution to compare a set of reference genes to the genes that occur in each module; in this case, our reference set was constrained to the genes included in the network analysis. A Bonferroni adjustment was applied within PANTHER to each GO term or pathway that occurred in the overrepresentation analysis. When many gene products within a module share a common GO term or are from the same annotated pathway, one can postulate that the module’s collective function is related to the ontology term or pathway. The statistical test for enrichment can produce a significant result if only one gene product from the module is associated with a GO term or pathway when the probability of even one gene product from the term/pathway appearing in the module by chance is extremely low. Because of this, we implemented an additional criterion that more than one gene product within the module needed to be associated with the term/pathway.

#### Phenotypic QTLs

To investigate the etiologic relationship between liver modules and genetic factors that may predispose animals to specific phenotypes, phenotypic QTLs were identified using the RGD’s QTL search. RGD contains a repository of genetic information for the rat species; as of 2015, the website contained records for 53,345 genes, 108,875 transcripts, and 2163 QTLs (Shimoyama et al. 2015). We considered all QTLs that were documented to overlap the peak of each module’s significant meQTL. We set a high standard for potentially relevant QTLs with a LOD score ≥10. Since this QTL data source is curated from published rat QTL papers, precise genome-wide *p* values are often not reported.

### Additional characterization of modules

#### Partial correlation

In the event that a module of interest had many gene products physically located within the same region of the genome, we assessed the biological nature of our findings with partial correlation. Associations between two co-localized gene products are more likely to share a biological function/process, if the partial correlation remains significant when accounting for an associated *cis*-locus (Supplementary Fig. S1). Pairwise correlations were recalculated between gene clusters, while controlling for each module’s meQTL. Connectivity and the number of relevant connections (*r* > |0.5|) were re-assessed under the partial correlation model.

#### Liver cell type-specific transcriptome analysis

Alcohol-naïve adult male BN-Lx/CubPrin and SHR/OlaPrin rats were shipped to the Integrative Liver Cell Core at University of Southern California for separation of liver cell types. Kupffer cells (KC) and hepatic stellate cells (HSC) were isolated by sequential digestion of rat liver with pronase and collagenase followed by low-speed centrifugation and discontinuous arabinogalactan gradient ultracentrifugation. This procedure has been described in detail by Kamimura and Tsukamoto (1995), and by Tsukamoto et al. (1995). Sinusoidal endothelial cells (SEC) were isolated by collagenase perfusion, density gradient centrifugation, and centrifugal elutriation as previously described by Deleve (1994). Primary hepatocyte cells (HC) were isolated aseptically according to the method of Moldéus et al. (1978). This method is based on collagenase digestion and separation of liver parenchymal cells. Fresh cells were shipped to the University of Colorado Denver for RNA extraction and microarray processing.

RNA from four rats per strain and cell type was extracted and hybridized to separate Affymetrix Rat Exon Arrays 1.0 ST (Affymetrix) in the same manner as described previously for the whole liver in the HXB/BXH panel. To make gene clusters comparable to our earlier analyses, the same methods used in the whole liver analysis were applied to the cell type-specific arrays; specifically, arrays were processed using the same probe mask and normalized with the same algorithm and batch corrections. A two-way ANOVA model was used to estimate expression means for each cell type and strain, and differential expression between cell types (either strain dependent or independent) was determined using an F-statistic and a false discovery rate (FDR) to correct for multiple comparisons. Further, genes were subset by module for pairwise cell type comparisons.

## Results

### Identification of RNA transcripts in the liver

#### Whole liver RNA sequencing

RNA isolated from each of the three livers of each strain (BN-Lx and SHR) was prepared and separately sequenced. We obtained 311 million paired-end reads for BN-Lx and 314 million for SHR. After trimming and the removal of reads that aligned to ribosomal RNA (rRNA), 224 million paired-end reads (72.0 %) from the BN-Lx rats aligned to the BN-Lx strain-specific genome and the synthetic spike-ins. 256 million paired-end reads (71.5 %) from the SHR aligned to the SHR strain-specific genome and the synthetic spike-ins. Synthetic spike-ins were included to improve normalization and batch correction.

#### Transcriptome reconstruction in progenitor strains

Within each progenitor strain transcriptome, an FPKM (based on combined reads within a strain) threshold of 1 was set to define a high-confidence transcript (Supplementary Fig. S2). For BN-Lx, we retained 17,343 transcripts (14 % of the total transcripts), with FPKM ranging from 1 to 11,952. Similarly in SHR, we retained 14,023 transcripts (16 % of the total transcripts), with FPKMs ranging from 1 to 5293.

After the high-confidence transcripts of the BN-Lx and SHR transcriptomes were combined and iteratively quantitated with CuffLinks, 18,260 transcripts (14,201 genes) remained; 6671 transcripts were annotated in the Ensembl database (Birney et al. 2004) and 11,589 were not included in the rn5 version of the Ensembl database (Table 1). It is important to note that using an RNA-Seq-derived transcriptome to select transcripts, we were able to identify many unannotated isoforms of annotated genes with respect to the Ensembl database,

**Table 1:** Summary of rat liver transcriptome reconstruction and RNA-Seq-guided microarray mask

Strain	Data type	Unique gene IDs	Total transcripts	Ensembl transcripts	Transcripts not annotated in the Ensembl database
BN-Lx	Complete transcriptome	123,143	143,107	28,836	114,271
	High-confidence transcripts	13,593	17,343	6998	10,345
SHR	Complete transcriptome	83,864	102,825	28,822	74,003
	High-confidence transcripts	10,321	14,023	7136	6887
BN-Lx & SHR	combined Transcriptome	14,201	18,260	6671	11,589
rn5	Affymetrix exon array gene clusters included in network analysis	9223	13,111	5994	7117

RNA-Seq high-confidence transcripts were required to have FPKM > 1 and length > 300 bp

### Quantitation of RNA expression in the livers of the HXB/BXH recombinant inbred panel

#### Microarray expression measurements

The same probe mask described by Saba et al. (2015) was used to filter the 4.1 million probes on the Affymetrix Rat Exon 1.0 ST Array to 3.8 million ‘high-integrity’ probes (890,607 probe sets). When these probe sets were mapped to the combined transcriptome of both the BN-Lx and SHR rats, 146,473 probe sets were contained entirely within liver transcripts that were identified from the RNA-Seq data. Specifically, 9847 gene products from the liver transcriptome were targeted by at least 1 probe set. All probe sets targeting a singular gene product were summarized into a gene cluster. After filtering out gene clusters that did not meet our detection above background criteria, 9223 gene clusters remained in our analysis (Table 1).

### Selection of genetically driven modules

Using the 9223 gene clusters, we identified 50 co-expression modules: each contained between 5 and 3369 gene products (median module size = 22.5). We calculated an eigengene for each of the modules to represent the expression of genes within each module. The eigengenes accounted for between 43 and 81 % of the variance within each module (median 58 %). Fourteen modules had significant meQTL genome-wide LOD scores, based on a genome-wide *p* value <0.05, and all of the eigengenes for these modules explained at least 57 % of the respective module variance (Table 2).

**Table 2:** Genetically driven co-expression modules in rat liver

Module	Genes		Eigengene	meQTL		
	Number of genes in module	Number of Ensembl annotated genes	Proportion of variance in module explained by eigengene	Location	LOD	Genome-wide *p* value
Thistle	19	14	0.56	Chr1: 177 Mb	8.47	0.004
Yellow3	15	8	0.60	Chr1: 282 Mb	8.65	0.004
Plum	23	11	0.60	Chr2: 223 Mb	6.67	0.016
Palevioletred2	18	10	0.58	Chr2: 248 Mb	5.69	0.008
Indianred3	10	6	0.58	Chr5: 155 Mb	4.54	0.048
Lightcyan	119	67	0.58	Chr6: 6.6 Mb	3.28	0.041
Firebrick4	45	17	0.59	Chr8: 7.4 Mb	5.06	0.005
Plum2	38	22	0.67	Chr10: 26 Mb	4.47	0.017
Lightpink2	5	3	0.81	Chr10: 67.2 Mb	8.50	0.009
Tan4	9	3	0.67	Chr10: 87 Mb	7.51	<0.001
Magenta3	5	3	0.75	Chr15: 107 Mb	8.48	0.004
Pink4	13	6	0.57	Chr16: 36 Mb	6.11	0.005
Lightblue4	11	7	0.64	Chr18: 53.8 Mb	7.85	0.007
Thistle2	37	21	0.58	Chr20: 8.0 Mb	6.39	0.004

Fourteen modules had significant module eigengene QTLs (meQTLs; genome-wide *p* < 0.05), and a proportion of variance in the module explained by the eigengene greater than 0.5. Although partial correlation was performed for the thistle2 module; these numbers reflect the number of genes that occurred prior to partial correlation

### Identifying biological relevance of modules

Seven of the modules contained at least ten Ensembl annotated gene products and, of these, four of the genetically driven modules were significantly enriched (adjusted *p* value <0.05) for at least one GO term or PANTHER pathway: thistle2, plum2, firebrick4, and lightcyan. To find physiological or behavioral traits associated with these modules, we used the RGD database to identify physiological and behavioral QTLs that overlapped the meQTLs of our significantly enriched modules (Table 3).

**Table 3:** Genetically driven liver co-expression modules with inferred biological relevance

Enrichment					
Module	GO term/PANTHER pathway	Smallest *p* value	Name	LOD	References
Thistle2	Antigen processing and presentation^a^, antigen processing and presentation of peptide or polysaccharide via MHC-class II, cellular defense response, MHC protein complex, Antigen binding	9.80 × 10^−17^	CIA autoantibody	39.9	Furuya et al. (2000)
			Food consumption	20.7	Marissal-Arvy et al. (2014a)
			Serum corticosterone level	20.5	Marissal-Arvy et al. (2014b)
			Adjuvant-induced arthritis	18.0	Kawahito et al. (1998), Joe 2006
Plum2	Immune response^a^	4.16 × 10^−2^	Food consumption	18.7	Marissal-Arvy et al. (2014a)
			Insulin level	10.5	Marissal-Arvy et al. (2014b)
Lightcyan	Receptor binding^a^, G protein-coupled receptor activity, actin binding	1.67 × 10^−3^	Bone mineral density	11.7	Koller et al. (2008)
			Bone mineral density	11.2	Koller et al. (2008)
Firebrick4	Vesicle-mediated transport^a^, protein transport	2.88 × 10^−3^	Insulin/glucose ratio	18.5	Marissal-Arvy et al. (2014b)

Summary of the results obtained from two public resources: PANTHER Gene List Analysis for PANTHER pathway and GO term enrichment, and the Rat Genome Database QTL search

^a^Marks the enrichment category that corresponds to the smallest *p* value

#### Thistle2

Twenty-three of the thistle2 module’s 37 gene products (Supplementary Table 1) were associated with aspects of the immune system according to gene ontology, and the module was highly enriched for several GO terms that are related to the immune system. Ten gene products from thistle2 were associated with antigen processing and presentation (GO: 0019882); this represents a 111-fold enrichment for this category (adjusted *p* value = 9.80 × 10^−17^). Of those ten gene products, two were specifically associated with antigen processing and presentation of peptide or polysaccharide antigen via MHC-class II (GO: 0002504) representing 100-fold enrichment for the category (adjusted *p* value = 3.13 × 10^−2^).

**Fig. 2:**
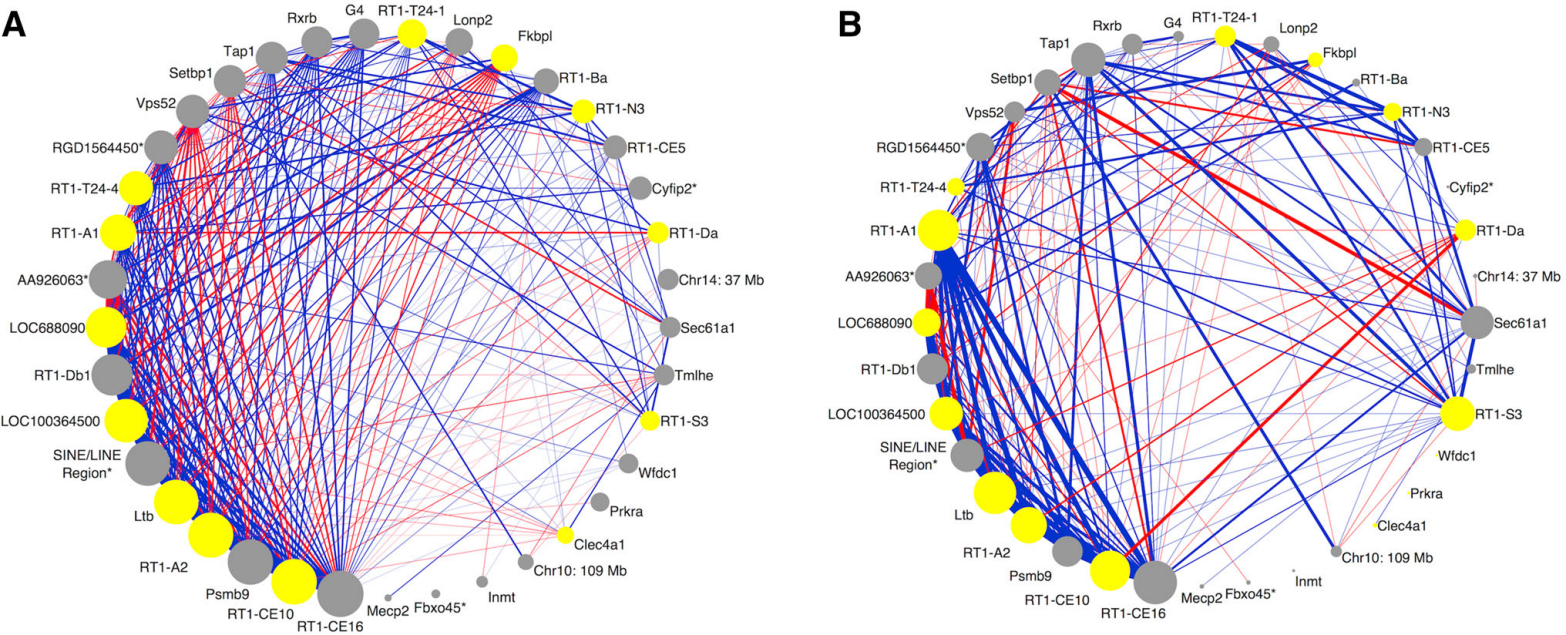
Connectivity within the thistle2 module. **a** Initial pairwise connections between gene products in the thistle2 co-expression module. **b** Pairwise connections that remained after adjustment for the module eigengene QTL, i.e., potential shared cis eQTL among genes. A red edge represents a negative correlation between the two nodes and a blue edge represents a positive correlation between two nodes. The thickness of each edge represents the strength of each correlation (i.e., a correlation with a larger magnitude has a thicker line), and edges are only visible if their associated correlation is greater than |0.60|. Unannotated genes are represented by chromosomal location. Genes are marked with an *asterisk* if none of its associated high-confidence transcripts were annotated in the Ensembl database, but at least one is similar to an annotated transcript from the rat Ensembl database, the rat RefSeq database, or the RefSeq database from other species. *Yellow nodes* represent gene products that were involved in module enrichment (immune system, antigen processing and presentation, cellular defense response, MHC protein complex, and antigen binding). RT-CE16, a Class I Major Histocompatibility Complex, is the hub gene product both before (**a**) and after (**b**) adjustment for shared cis eQTL

The thistle2 module was also enriched for several other biological, cellular, and molecular gene ontology terms. Under biological processes, four genes were associated with cellular defense response (GO: 0006968), representing 22-fold enrichment for the category (adjusted *p* value = 5.50 × 10^−3^). For the cellular component, two genes were associated with the MHC protein complex (GO0042611), representing a 200-fold enrichment (adjusted *p* value = 3.64 × 10^−3^). Finally, with respect to molecular function, eight genes were associated with antigen binding (GO: 0003823), representing a 133-fold enrichment (adjusted *p* value = 2.17 × 10^−13^). Gene products that are involved in these processes are represented as yellow nodes (circles) in Fig. 2.

The top five most highly connected gene products in this module function within the immune system. The hub is an RT1-class 1 gene product (RT1-CE16) that functions as an MHC-class I molecule. The remaining gene products include 2 other MHC-class I-related molecules, a gene product that processes MHC-class I molecules, and the lymphotoxin beta gene (TNF superfamily, member 3).

A total of 24 phenotypic QTLs overlapped thistle2’s meQTL; four of these met our criteria: collagen-induced arthritis autoantibody (Ciaa1), adjuvant-induced arthritis (Aia1), food consumption (Foco23), and serum corticosterone levels (Scort12).

Two of the overlapping phenotypic QTLs are associated with autoimmunity. The collagen-induced arthritis autoantibody (Ciaa1) phenotype is the IgG autoantibody titer against native rat type II collagen (Furuya et al. 2000). This phenotype is a model for rheumatoid arthritis; rats with higher susceptibility to collagen-induced arthritis have higher levels of the IgG autoantibody, which is a biomarker for the autoimmune disease. Adjuvant-induced arthritis is another model for rheumatoid arthritis. The adjuvant-induced arthritis phenotype (Aia1) is associated with disease severity; measures of the arthritis index were used to determine severity of the disease (Joe 2006; Kawahito et al. 1998).

The remaining QTLs were associated with food preference and basal corticosterone levels. The food consumption phenotype (Foco23) is a reflection of macronutrient selection and weight gain. Marissal-Atvy et al. (2014a) allowed rats to self-select from different macronutrient-driven diets (i.e., protein-, carbohydrate-, and fat-rich diets), and documented diet preference and the resulting weight gain. This phenotypic QTL is associated with carbohydrate preference in female rats that have differential macronutrient preferences. Marissal-Atvy et al. (2014b) were also interested in phenotypes that were related to genetic variability within the hypothalamic–pituitary–adrenocortical (HPA) axis, so they investigated the overlap between QTLs associated with the function of the HPA and phenotypes that were associated with carbohydrate metabolism. One of these metabolism-related phenotypes was basal state serum corticosterone levels (Scort12).

#### Plum2

**Fig. 3:**
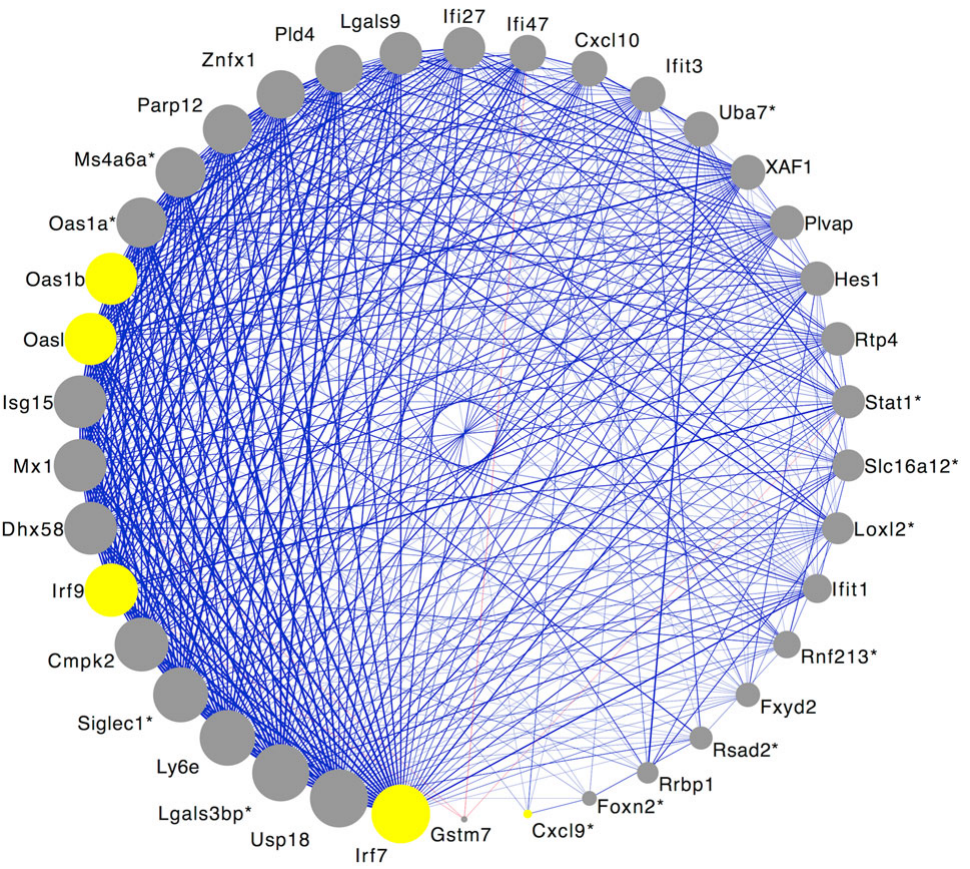
Connectivity within the plum2 module. Initial pairwise connections between gene products in the plum2 co-expression module. A *red edge* represents a negative correlation between two nodes and a *blue edge* represents a positive correlation between two nodes. The thickness of each edge represents the strength of each correlation (i.e., a correlation with a larger magnitude has a *thicker line*), and edges are only visible if their associated correlation is greater than |0.60|. Genes are marked with an *asterisk* if none of its associated high-confidence transcripts were annotated in the Ensembl database, but at least one is similar to an annotated transcript from the rat Ensembl database, the rat RefSeq database, or the RefSeq database from other species. *Yellow nodes* represent gene products that were involved in module enrichment (immune response). Irf7, Interferon regulatory factor 7, is the hub gene product

There were 38 gene products in the plum2 module (Supplementary Table 2), and 22 of those gene products were Ensembl annotated. The module hub gene product was interferon regulatory factor 7 (Irf7), and at least 14 gene products within the plum2 module were related to interferon regulation, response to various types of interferon, defense to virus, and immune response. The module is significantly enriched for the immune response (GO: 0006955); it had a 13-fold enrichment for the category (adjusted *p* value = 4.16 × 10^−2^). Gene products that are involved in these processes are represented as yellow nodes (circles) in Fig. 3.

We identified 35 phenotypic QTLs that overlapped the plum2 module’s meQTL; 2 of these phenotypic QTLs met our criteria: food consumption (Foco17) and plasma insulin level (Insul27). Like Foco23 (food consumption phenotype in thistle2), the food consumption phenotype overlapping plum2 (Foco17) is also associated with carbohydrate preference in female rats. While investigating the association between the HPA and metabolism, Marissal-Atvy et al. (2014b) found the plasma insulin level QTL (Insul27).

#### Lightcyan

There are 119 gene products in the lightcyan module; 67 gene products were Ensembl annotated. The hub gene product is prostaglandin G/H synthase 1 (Ptgs1), an oxygenase involved in inflammation mediated by chemokine and cytokine signaling pathways. The module was enriched for several GO terms that involved binding. Five gene products were associated with actin binding (GO: 0003779), representing a ninefold enrichment for the category (adjusted *p* value = 2.68 × 10^−2^). Ten gene products were associated with receptor binding (GO: 0005102), with sixfold enrichment for the category (adjusted *p* value = 1.67 × 10^−3^). Finally, four genes were associated with G protein-coupled receptor activity (GO: 000490), with 17-fold enrichment for the category (adjusted *p* value = 1.67 × 10^−2^).

We identified 16 phenotypic QTLs that overlapped lightcyan’s meQTL; 2 of these phenotypic QTLs met our criteria, and both were related to bone mineral density (Bmd51 and Bmd52). The bone mineral density phenotypes Bmd51 and Bmd52 are associated with the variability in femur areal and volumetric bone mineral density, respectively (Koller et al. 2008).

#### Firebrick4

There were 45 gene products in the firebrick4 module, and 17 of them were annotated in the Ensembl database. The hub was the coatomer protein complex subunit alpha gene product (Copa), which is a vesicle coat protein.

Firebrick4 was enriched for several GO term transport categories. There were seven gene products associated with vesicle-mediated transport (GO: 0016192), representing an eightfold enrichment for the category (adjusted *p* value = 2.88 × 10^−3^), and seven were associated with protein transport (GO: 0015031), with sixfold enrichment for the category (adjusted *p* = 1.98 × 10^−2^).

Five phenotypic QTLs overlapped the firebrick4 meQTL, but only one met our criteria: insulin/glucose ratio (Insglur5), which is associated with insulin resistance. In the same study that was referenced for the plasma insulin QTL that overlapped the plum2 module, Marissal-Atvy et al. (2014b) identified insulin resistance as a relevant phenotype for metabolism. Insulin resistance was calculated by finding the percent decrease in glycaemia after injecting animals with insulin.

Agreement was found between the PANTHER enrichment and RGD QTL databases for the thistle2 and plum2 modules (Table 3). There were strong similarities between gene product enrichment and overlapping phenotypic QTLs for the thistle2 module. In both databases, there was strong evidence that this module is related to the immune system. The strongest overlapping phenotypic QTL was related to the IgG autoantibody and autoimmune disease, while the module was enriched for the immune system, and antigen presentation and processing. The similarities between enrichment and overlapping phenotypic QTLs are not as obvious for the plum2 module; however, several of the proteins that directly impact food consumption and insulin regulation are associated with pro-inflammatory responses (Wensveen et al. 2015), and the module was enriched for genes related to immune response. With these similarities in mind, we chose to further analyze our results with the thistle2 and plum2 gene product networks.

### Additional characterization of modules

#### Partial correlation

Twenty-five of the gene products within the thistle2 module were co-localized to chromosome 20, so we performed partial correlation analysis to determine if the correlation among gene products was due to independent causal loci in linkage disequilibrium. After adjusting for the meQTL, the module retained the majority of its nodes; in fact, none of gene products associated with chromosome 20 lost all of their connections (Fig. 2b). This indicates that these gene products from chromosome 20 likely have a biological relationship, and are not simply expressed in similar manner because of linkage disequilibrium among causal loci.

#### Liver cell type-specific transcriptome analysis

Of the 9331 genes that remained after pre-processing of the cell type-specific arrays, 8447 (90 %) were differentially expressed among cell types (FDR < 0.05). The normalized expression data and the original CEL files are available on PhenoGen. Because the thistle2 and plum2 modules are related to the immune system and immune response, we compared the Kupffer cell expression of genes within the modules to each of the other three cell types in this cell-specific analysis (hepatocytes, hepatic stellate cells, and sinusoidal endothelial cells).

##### Thistle2

**Fig. 4:**
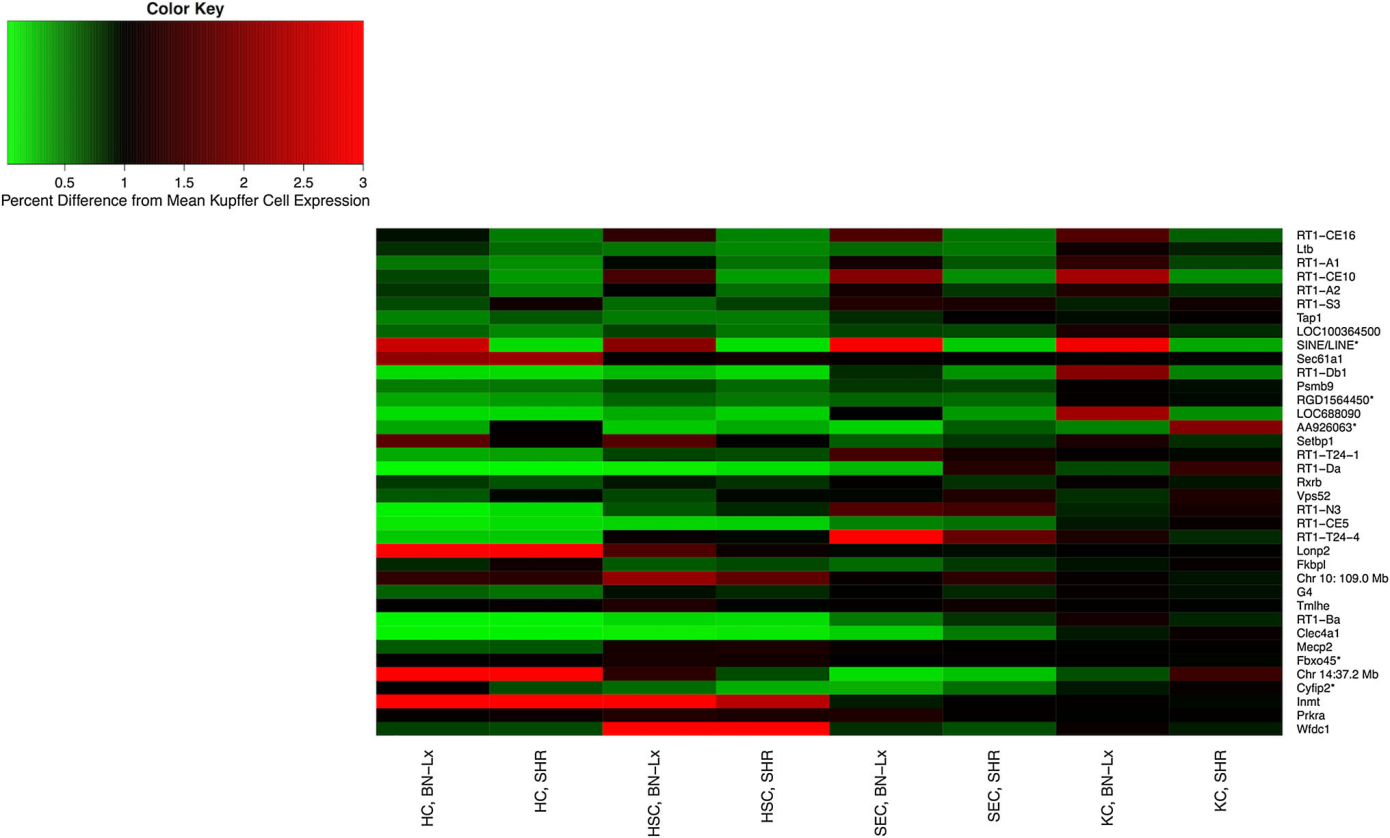
Cell type-specific expression of gene products in the thistle2 module. Cell type: *HC* hepatocyte cells, *HSC* hepatic stellate cells, *SEC* sinusoidal endothelial cells, *KC* Kupffer cells. Strain: *BN-Lx* Brown Norway with polydactyly-luxate mutation, *SHR* spontaneous hypertensive rat. The color and intensity of each cell represent its relative expression compared to the strain average in the Kupffer cells, e.g., a value of 2 indicates that the expression in that strain and cell type is twice the average expression in the Kupffer cells. Genes are in order of connectivity after partial correlation, with the highest connectivity at the *top* of the graphic and the lowest connectivity at the *bottom*

Of the 37 gene products in the thistle2 module, Kupffer cells had the highest expression among the 4 cell types in 27 (57 %) of the gene products (Fig. 4). The hub gene, RT1-CE16, was up-regulated in the Kupffer cells; these cells showed 43 % higher expression of the hub gene compared to hepatocytes, 23 % higher expression compared to hepatic stellate cells, and 8 % higher expression compared sinusoidal epithelial cells. The five most highly connected genes of the thistle2 module were up-regulated in Kupffer cells, and of the top 15 most highly connected genes in the thistle2 module, 13 were up-regulated in Kupffer cells.

##### Plum2

**Fig. 5:**
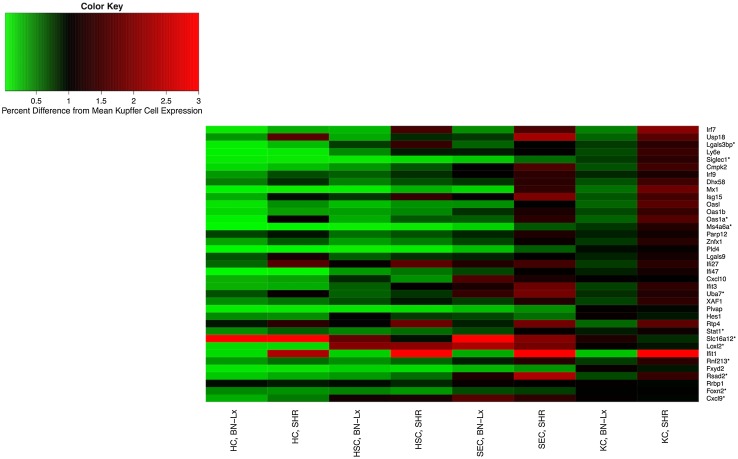
Cell type-specific expression of gene products in the plum2 module. Cell type: *HC* hepatocyte cells, *HSC* hepatic stellate cells, *SEC* sinusoidal endothelial cells, *KC* Kupffer cells. Strain: *BN-Lx* Brown Norway with polydactyl luxate mutation, *SHR* spontaneous hypertensive rat. The color and intensity of each cell represent its relative expression compared to the strain average in the Kupffer cells, e.g., a value of 2 indicates that the expression in that strain and cell type is twice the average expression in the Kupffer cells. Genes are in order of connectivity, with the highest connectivity at the *top* of the graphic and the lowest connectivity at the *bottom*

Of the 38 gene products in the plum2 module, Kuppfer cells had the highest expression among the four cell types in 17 (45 %) of the gene products (Fig. 5). The hub gene, Ifr7, was up-regulated in Kupffer cells; these cells had 520 % higher expression of the hub gene compared to hepatocytes, 54 % higher expression compared to hepatic stellate cells, and 19 % higher expression compared to sinusoidal epithelial cells.

Although not discussed here, the cell type-specific expression values for gene products in the firebrick4 and lightcyan modules are displayed in Supplementary Figs. 3 and 4.

## Discussion

The approach discussed in this paper included increasing the interpretability of microarray data using RNA-Seq and DNA-Seq data, constructing gene product networks with WGCNA, and applying stringent criteria for the identification of genetically regulated modules within the rat liver. This resulted in the thistle2 module with gene products that were related to the immune system, including the hub gene product RT1-CE16 (an MHC-class I molecule). Thistle2 was also enriched for the immune system process, and for antigen processing and presentation. Not surprisingly, the majority of gene products in this module had higher expression in Kupffer cells than in hepatocytes, hepatic stellate cells, or sinusoidal endothelial cells (Figs. 4, 5). Further, after searching through publicly available phenotypic QTLs that overlapped the thistle2 meQTL, we found two phenotypic QTLs that mapped to the same region and were associated with immune system function: collagen-induced arthritis (Ciaa1) and adjuvant-induced arthritis (Aia1). Interestingly, Ciaa1 is located at the marker for lymphotoxin alpha, and the thistle2 module contains the gene product for lymphotoxin beta; lymphotoxin alpha and beta specifically bind to each other.

The collagen-induced and adjuvant-induced arthritis rat models are commonly used to study rheumatoid arthritis (Joe 2006). Cell type-specific analyses showed that the majority of components within the thistle2 module primarily reside in Kupffer cells. These cells are the monocyte and macrophage cells of the liver (Li et al. 2010), are responsible for cytokine production and the presentation of antigen to B and T cells (Li et al. 2010), and are involved in systemic immune tolerance (You et al. 2008; Xu et al. 2014). It has also been shown that these monocyte and macrophage systems are an essential component of autoimmune disease (Li et al. 2010). With all of these in mind, it can be postulated that the liver contributes to rheumatoid arthritis through a genetically influenced and Kupffer cell-mediated effect on systemic immune tolerance that predisposes to, or diminishes, the likelihood of autoimmune disease.

Behavioral phenotypic QTLs for food consumption and corticosterone levels also overlapped the thistle2 meQTL. Interestingly, factors influencing food consumption have been associated with measures of immune function. Leptin released from adipose tissue regulates the behavior of food consumption by suppressing appetite, and low levels of leptin have been shown to also suppress the immune system (Fernández-Riejos et al. 2008). It has also been observed that patients with rheumatoid arthritis have higher levels of leptin, and that leptin-deficient mice are less susceptible to antigen-induced arthritis (Lago et al. 2007). This implies that leptin, which plays an important role in food consumption, may also play a role in immune suppression and autoimmunity. Finally, food consumption and circulating corticosterone levels have been linked. In rats, it has been shown that the daily pattern of corticosterone can be modified by restrictive feeding (Namvar et al. 2016), and that high levels of glucocorticoids increase the intake of foods with high fat and/or sucrose (Dallman et al. 2005).

We also identified the plum2 module, which was enriched for immune response. It contained gene products associated with the pro-inflammatory response: peg interferon alpha-2a/b, chemokines, interferon regulatory factors, and response to interferon. Additionally, many of the gene products in this module showed higher expression in Kupffer cells, when compared to the other three cell types. Phenotypic QTLs for food consumption and insulin regulation overlapped the genetic control of expression levels in plum2. Cortisol provides an important link between food consumption and insulin regulation; high cortisol levels can promote insulin resistance at the cellular level, which increases the hunger signals that lead to overeating (Aronson 2009). Leptin also helps to explain the relationship between food consumption and inflammation, because it functions in part as a pro-inflammatory cytokine and its levels are increased by inflammatory stimuli such as IL-1 and IL-6 (Lago et al. 2007). It has also been postulated that there is a relationship between insulin resistance, interferon-gamma, and inflammation (Wensveen et al. 2015). Wensveen et al. showed that obesity stimulates the up-regulation of interferon-gamma and NK cell proliferation, which in turn increases the production of pro-inflammatory macrophages and insulin sensitivity.

In this analysis, we identified two genetically driven modules with obvious biological relevance; however, this does not imply that the remaining 48 modules do not represent biological pathways. Our goal was to demonstrate how these methods and models, in conjunction with currently available public databases, could be used to uncover biological relevance and, indeed, we identified two liver modules associated with immune response. There are several possible reasons why more modules were not linked to biological pathways. Nearly 5000 of the RNA-Seq-derived liver genes were not interrogated by the array, and some of the genes that were interrogated by the array failed to be detected above background; consequently, these technology-related limitations could miss important genes. Thus, modules that are derived in this manner may not include every gene product that is relevant to the related pathway; instead, they represent a set of the measureable genes related to the pathway.

Publication biases, associated with the public databases used to define biological relevance, are another possible limitation for identifying more biologically relevant modules. For example, the QTL database may be biased to phenotypes that have been researched with respect to genetic determinants. Some phenotypes may have a higher abundance of QTLs, because rats have been good models for studying a particular disease process (e.g., alcohol response, blood pressure, arthritis); consequently, QTLs are both overrepresented and underrepresented in the current databases.

Further, regardless of the rat population used for mapping, all of the RGD QTLs were considered. Although this “inclusive” approach may identify phenotypic QTL that do not represent the same causal loci as the overlapping meQTL, because they were detected in two different populations, the two loci are likely in linkage disequilibrium and may have similar effects. It is true that without detailed QTL studies using rat populations derived from the same or similar strains as the progenitors of the HXB/BXH RI panel, we may miss relevant phenotypic QTL.

Some may argue that using a heterogeneous tissue sample will ‘mask’ important transcription patterns. Although some connections between genes will be missed, the connections that are identified are robust strong signals that are important in a systems genetics analysis. When we examined cell type-specific expression in modules that were associated with immunity and inflammation, as predicted, both modules contained strong signals from Kupffer cells. Kupffer cells account for a small proportion of the total cells in healthy liver, but we were still able to recover relevant pathways that they likely influence. Using whole tissue is not an end-all approach, but rather a starting point to identify key components of a system/network.

In conclusion, our systems genetics approach improved the interpretability of microarray data by mapping probes to progenitor strain-specific transcriptomes, and allowed for the identification and characterization of liver co-expression modules within our panel of rats. This combination of methods and data allowed us to characterize previously unannotated gene products, and to identify genetically and biologically relevant gene networks. RNA-based networks can serve many purposes in future research: (1) they can be viewed as an intermediate product between DNA sequence variants and a particular phenotype, (2) they can be used to “hypothesize” about the function of individual unannotated transcripts through associations within a module, and (3) they can be used to identify optimal animal strains for future studies of disease etiology by prediction of individual strain predisposition to, magnitude of, and progression of disease.

## Electronic supplementary material

Below is the link to the electronic supplementary material.

Supplementary material 1 (DOCX 354 kb)
