# Supplementary material for: Uncovering the liver’s role in immunity through RNA co-expression networks
Source: Mamm Genome. 2016 Jul 11;27(9):469–84. doi: 10.1007/s00335-016-9656-5 (PMC5002042; doi:10.1007/s00335-016-9656-5)
Supplement: Supplementary file 1 — Supplementary material 1 (DOCX 354 kb) [file 335_2016_9656_MOESM1_ESM.docx]

Supplementary Table 1: Genes of the thistle2 “Immunoglobulin” module. Gene function was extracted from RGD. Connectivity represents the sum of the absolute values of all pairwise correlation coefficients associated with the particular gene. Connections represents the number of pairwise correlations > |0.5| that are associated with the gene of interest. Genes are marked with an asterisk (*) if none of its associated high-confidence transcripts were annotated in the Ensembl database, but at least one is similar to an annotated transcript from the rat Ensembl database, the rat RefSeq database, or the RefSeq database from other species. Abbreviations: Chromosome (Chr), Megabase (Mb), Major Histocompatibility Complex (MHC).

| Gene Symbol | Gene Name | Function | Location | Connectivity | Connections | Connectivity after partial correlation | Connections after partial correlation |
| --- | --- | --- | --- | --- | --- | --- | --- |
| RT1-A1 | RT1 class Ia, locus A1 | MHC Class I | Chr 20: 7.5 Mb | 23.5 | 27 | 18.3 | 19 |
| RT1-CE16 | RT1 class I, locus CE16 | MHC Class I | Chr 20: 5.9 Mb | 27.2 | 33 | 18.1 | 21 |
| Ltb | lymphotoxin beta (TNF superfamily, member 3) | Tumor Necrosis Factor | Chr 20: 6.9 Mb | 26.6 | 33 | 17.9 | 21 |
| RT1-CE10 | RT1 class I, locus CE10 | MHC Class I | Chr 20: 0.16 Mb | 26.9 | 34 | 17.6 | 19 |
| RT1-A2 | RT1 class Ia, locus A2 | MHC Class I | Chr 20: 7.4 Mb | 26.8 | 34 | 17.0 | 17 |
| Psmb9 | proteasome (prosome, macropain) subunit, beta type, 9 | Processes Class I MHC Peptides | Chr 20: 6.1 Mb | 26.8 | 34 | 16.7 | 14 |
| LOC100364500 | RT1 class I, locus CE11-like | MHC Class I | Chr 20: 4.8 Mb | 26.2 | 32 | 16.5 | 15 |
| SINE/LINE* |  | Uncharacterized | Chr 20: 0.17 Mb | 26.5 | 32 | 16.3 | 14 |
| Tap1 | transporter 1, ATP-binding cassette, sub-family B | ATP-binding Cassette Transporter | Chr 20: 6.1 Mb | 21.9 | 25 | 15.2 | 17 |
| RGD1564450* | RGD1564450 - like | Interacts with 6-propyl-2-thiouracil; bisphenol A; cadmium dichloride (inferred) | Chr20: 9.3 Mb | 22.5 | 25 | 15.2 | 14 |
| RT1-S3 | RT1 class Ib, locus S3 | MHC Class I | Chr 20: 5.3 Mb | 17.3 | 17 | 15.1 | 18 |
| RT1-Db1 | RT1 class II, locus Db1 | MHC Class II | Chr 20: 6.2 Mb | 25.3 | 29 | 14.4 | 14 |
| Sec61a1 | Sec61 alpha 1 subunit | Membrane Traffic | Chr 4: 185.7 Mb | 17.7 | 19 | 14.2 | 17 |
| LOC688090 | similar to RT1 class II histocompatibility antigen, B-1 beta chain precursor | MHC Class II | Chr 20: 6.1 Mb | 25.1 | 29 | 14.2 | 13 |
| AA926063* | AA926063gene - like | Integral component of membrane (inferred) | Chr 20: 7.5 Mb | 24.2 | 24 | 13.7 | 13 |
| Setbp1 | SET binding protein 1 | Methyltransferase;DNA Binding Protein | Chr 18: 74.8 Mb | 22.0 | 26 | 13.3 | 13 |
| Lonp2 | lon peptidase 2, peroxisomal | ATP-binding and Peptidase Activity; Protease Binding | Chr 19: 3.2 Mb | 20.1 | 25 | 13.1 | 8 |
| RT1-Da | RT1 class II, locus Da | MHC Class II | Chr 20: 6.2 Mb | 17.9 | 18 | 12.7 | 11 |
| RT1-T24-1 | RT1 class I, locus T24, gene 1 | MHC Class I | Chr 20: 5.4 Mb | 20.8 | 23 | 12.4 | 10 |
| Rxrb | retinoid X receptor beta | Mediates Retinoic Acid | Chr 20: 5.9 Mb | 21.5 | 24 | 12.4 | 11 |
| RT1-N3 | RT1 class Ib, locus N3 | MHC Class I | Chr 20: 5.3 Mb | 18.8 | 18 | 12.1 | 8 |
| RT1-T24-4 | RT1 class I, locus T24, gene 4 | MHC Class I | Chr 20: 5.3 Mb | 22.7 | 26 | 12.1 | 9 |
| RT1-CE5 | RT1 class I, locus CE5th | MHC Class I | Chr 20: 7.0 Mb | 18.8 | 22 | 11.8 | 8 |
| Vps52 | vacuolar protein sorting 52 homolog | Membrane Traffic | Chr 20: 7.5 Mb | 22.3 | 22 | 11.6 | 10 |
| Fkbpl | FK506 binding protein-like | Isomerase;Chaperone;Calcium-binding protein | Chr 20: 6.5 Mb | 20.0 | 20 | 11.5 | 7 |
| Gene.135 |  | Uncharacterized | Chr 10: 109.0 Mb | 15.8 | 15 | 11.0 | 5 |
| G4 | G4 protein | Uncharacterized Protein | Chr 20: 7.2 Mb | 21.3 | 22 | 10.4 | 5 |
| Tmlhe | trimethyllysine hydroxylase, epsilon | Trimethyllysine Dioxygenase Activity; Oxidoreductase Activity | Chr 20: 231.3 kb | 17.6 | 18 | 8.6 | 4 |
| Clec4a1 | C-type lectin domain family 4, member A1 | Cell Adhesion | Chr 4: 223.0 Mb | 16.3 | 15 | 8.2 | 1 |
| Gene.15320 |  | Ambiguous, only one exon of the multi-exon gene Ociad2 | Chr 14:37.2 Mb | 17.8 | 20 | 7.5 | 1 |
| RT1-Ba | RT1 class II, locus Ba | MHC Class II | Chr 20: 6.1 Mb | 19.4 | 18 | 7.5 | 3 |
| Mecp2 | methyl CpG binding protein 2 | Chromatin DNA Binding; Enzyme Binding | Chr 1: 152.5 Mb | 12.5 | 3 | 7.3 | 1 |
| Fbxo45* | F-box protein 45 -like | F-box protein (inferred) | Chr 11: 74.9 Mb | 13.1 | 8 | 6.5 | 1 |

| **Supplementary Table 2: Genes of the plum2 “Interferon and Defense” Module.** Gene function was extracted from RGD. Connectivity represents the sum of the absolute values of all pairwise correlation coefficients associated with the particular gene. Connections represents the number of pairwise correlations > \|0.5\| that are associated with the gene of interest. Genes are marked with an asterisk (*) if none of its associated high-confidence transcripts were annotated in the Ensembl database, but at least one is similar to an annotated transcript from the rat Ensembl database, the rat RefSeq database, or the RefSeq database from other species. Abbreviations: Chromosome (Chr), Megabase (Mb).**Gene Symbol** | Gene Name | Function | Location | Connectivity | Connections |
| --- | --- | --- | --- | --- | --- |
| Irf7 | Interferon regulatory factor 7 | DNA binding, immunoglobulin response, interferon production | Chr 1: 214 Mb | 29.6 | 35 |
| Usp18 | Ubiquitin specific peptidase 18 | Protease | Chr 4: 154 Mb | 29.4 | 36 |
| Lgals3bp* | Lectin, galactoside-binding, soluble, 3 binding protein - like | Scavenger receptor activity, cell adhesion, receptor mediated endocytosis (inferred) | Chr 10: 107 Mb | 29.2 | 34 |
| Ly6e | Lymphocyte antigen 6 complex, locus E | Adrenal gland development, epinephrine secretion, in utero embryonic development | Chr 7: 116 Mb | 28.8 | 37 |
| Siglec1* | Sialic acid binding Ig-like lectin 1, sialoadhesin - like | Carbohydrate binding, apoptotic processes (inferred) | Chr 3: 130 Mb | 28.7 | 35 |
| Cmpk2 | Cytidine monophosphate kinase 2, mitochondrial | Exhibits thymidylate kinase activity | Chr 6: 46 Mb | 28.4 | 36 |
| Irf9 | Interferon regulatory factor 9 | Core promoter binding, interferon | Chr 15: 34 Mb | 28.3 | 36 |
| Dhx58 | DEXH box polypeptide 58 | RNA binding, zinc ion binding | Chr 10: 89 Mb | 28.2 | 35 |
| Mx1 | Myxovirus resistance 1 | GTP binding, immune response | Chr 11: 38 Mb | 28.2 | 36 |
| Isg15 | ISG15 ubiquitin-like modifier | Protein tag, defense response to bacterium and virus | Chr 5: 174 Mb | 28.1 | 35 |
| Oas1b | 2'-5'-oligoadenylate synthase 1F | nucleotidyltransferase;nucleic acid binding;defense/immunity protein, defense to virus | Chr 12: 41 Mb | 28 | 37 |
| Oasl | 2'-5'-oligoadenylate synthase-like protein 1 | nucleotidyltransferase;nucleic acid binding;defense/immunity protein, negative regulation of viral genome replication | Chr 12: 47 Mb | 28 | 32 |
| Ms4a6a* | Membrane-spanning 4-domains, subfamily A, member 6A - like | Integral component of membrane (inferred) | Chr 1: 235 Mb | 27.6 | 35 |
| Oas1a* | 2'-5'-oligoadenylate synthase 1A - like | Response to interferon-alpha and virus, glucose homeostasis (inferred) | Chr 12: 43 Mb | 27.6 | 33 |
| Parp12 | Poly (ADP-ribose) polymerase family, member 12 | nucleic acid binding | Chr 4: 67 Mb | 27.3 | 33 |
| Znfx1 | NFXX1-Type Zinc finger containing protein 1 | DNA helicase;RNA helicase;endoribonuclease;helicase;nuclease;hydrolase | Chr 3: 164 Mb | 27.1 | 34 |
| Pld4 | Phospholipase D4 | Catalytic activity | Chr 6: 137 Mb | 27 | 34 |
| Lgals9 | Lectin, galactoside-binding, soluble 9 | Carbohydrate binding, enzyme binding, response to interferon and virus | Chr 10: 65 Mb | 25.7 | 33 |
| Ifi27 | Interferon alpha-inducible protein 27-like protein 2A | Lamin binding, RNA polymerase II activating transcription factor binidng | Chr 6: 127 Mb | 25.6 | 30 |
| Ifi47 | Interferon gamma-inducible protein 47 | GTP binding, defense response | Chr 10: 34 Mb | 24.4 | 31 |
| Cxcl10 | C-X-C motif chemokine 10 | Chemokine, response to heat, immune response, heparin binding | Chr 14: 17 Mb | 24.2 | 34 |
| Ifit3 | Interferon-induced protein with tetratricopeptide repeats 3 | Identical binding, response to interferon-beta, regulation of apoptotic process and cell proliferation | Chr 1: 253 Mb | 24.1 | 31 |
| Uba7* | Ubiquitin-like modifier activating enzyme 7 - like | ISG15 activating enzyme activity, protein modification (inferred) | Chr 8: 116 Mb | 24.1 | 34 |
| XAF1 | XIAP-associated factor 1 | Regulation of protein complex assembly, response to interferon-beta | Chr 10: 59 Mb | 24 | 27 |
| Plvap | Plasmalemma vesicle-associated protein | Protein homodimerization, MAPK cascade, regulates cellular extravasation, TNF-mediated signaling pathway | Chr 16: 20 Mb | 23.9 | 30 |
| Hes1 | Hes family bHLH transcription factor 1 | Nucleotide binding, response to fatty acid, interleukin-1, nerve growth factor stimulus | Chr 11: 74 Mb | 23.8 | 30 |
| Stat1* | Signal transducer and activator of transcription 1 - like | Binding of CCR5 chemokine receptor, DNA, and protein phosphatase 2A (inferred) | Chr 9: 54.0 Mb | 23.6 | 29 |
| Rtp4 | Receptor transporting protein 4 | Detectin of chemical stimulus involved in sensory perception of bitter taste, protein targeting to membrane | Chr 11: 81 Mb | 23.6 | 30 |
| Loxl2* | Lysyl oxidase – like 2 - like | Binding of chromatin, methylated histones, oligosaccharides (inferred) | Chr 15: 55 Mb | 23.3 | 29 |
| Slc16a12* | Solute carrier family 16, member 12 - like | Creatine transmembrane transporter activity (inferred) | Chr 1: 260 Mb | 23.3 | 32 |
| Ifit1 | Interferon-induced protein with tetratricopeptide repeats 1 | Cellular response to interferon alpha, interferon-beta, virus | Chr 1: 253 Mb | 22.6 | 29 |
| Rnf213* | Ring finger protein 213 - like | ATPase activity, ubiquitin-protein transferase activity (inferred) | Chr 10: 108 Mb | 22.2 | 27 |
| Fxyd2 | FXYD domain-containing ion transport regulator 2 | cation transporter; ion channel; enzyme modulator; multiple pharmacodynamics pathways | Chr 8: 50 Mb | 21.5 | 28 |
| Rsad2* | Radical S-adenosyl methionine domain containing 2 | Ossicfication, CD4-positive, alpha-beta T cell activation` | Chr 6: 54.4 Mb | 21.2 | 24 |
| Rrbp1 | Ribosome-binding protein 1 | Poly-RNA binding, osteoblast differentation | Chr 3: 138 Mb | 20.8 | 26 |
| Foxn2* | Forkhead box N2 - like | Sequence specific DNA binding, transcription factor activity (inferred) | Chr 6: 22 Mb | 19.3 | 17 |
| Cxcl9* | Chemokine (C-X-C motif) ligand 9 - like | Chemokine activity (inferred) | Chr 14: 17 Mb | 17.8 | 12 |
| Gstm7 | Glutathione S-transferase Yb-3 | Glutathione transferase activity, cellular response to drug and organic cyclic compunds, phase I biotransformation via cytochrome P450 | Chr 2: 211 Mb | 17.3 | 12 |

**Supplementary Table 3: Genes of the firebrick4 module.** Gene function was extracted from RGD. Connectivity represents the sum of the absolute values of all pairwise correlation coefficients associated with the particular gene. Connections represents the number of pairwise correlations > |0.5| that are associated with the gene of interest. Genes are marked with an asterisk (*) if none of its associated high-confidence transcripts were annotated in the Ensembl database, but at least one is similar to an annotated transcript from the rat Ensembl database, the rat RefSeq database, or the RefSeq database from other species. Abbreviations: Chromosome (Chr), Megabase (Mb)

| Gene Symbol | Gene Name | Function | Location | Connectivity | Connections |
| --- | --- | --- | --- | --- | --- |
| Gfpt1* | Glutamine fructose-6-phosphate transaminase 1 | amino acid binding, carbohydrate binding, glutamine-fructose-6-phosphate transaminase | Chr 4: 183.4 | 30.8 | 39 |
| Gene.14214 |  |  | Chr 7: 24.8 Mb | 30.3 | 41 |
| Copa | Coatomer protein complex subunit alpha | structural moleule activity | Chr 13: 95.0 Mb | 30.1 | 37 |
| Hdlbp | High density lipoprotein binding protein | poly(A) RNA binding | Chr 9: 100.2 Mb | 29.4 | 37 |
| RGD1306487 | RAB3 GTPase activating protein subunit 1 | GTPase activator, Rab GTPase binding | Chr 13: 49.7 | 29.2 | 40 |
| Sec24d | SEC24 homolog D, COPII coat complex component | zinc ion binding | Chr 2: 246.9 Mb | 29.1 | 39 |
| Morf4l2 | Mortality factor 4-like protein 2 | striated muscle cell differentation, positive regulation of transcription from RNA polymerase II promoter | Chr X: 107.3 Mb | 29.1 | 39 |
| Ttpal* | Tocopherol (alpha) transfer protein-like | transporter activity | Chr 3: 166.2 Mb | 28.8 | 39 |
| Maged1 | MAGE family member D1 | transcription coactivator activity | Chr X: 64.7 Mb | 28.8 | 39 |
| Slc33a1 | Solute carrier family 33, member 1 | BMP signalling pathway, SMAD protein signal transduction | Chr 2: 173.9 Mb | 28.6 | 35 |
| Cxxc1 | CXXC finger 1 (PHD domain) | core promoter proximal region sequence-specific DNA binding, histone methyltransferase activity, unmethylated CpG binding | Chr 18: 69.3 Mb | 28.5 | 35 |
| Arcn1* | Archain 1 | poly(A) RNA binding | Chr 8: 47.7 Mb | 28.4 | 38 |
| Adar | Double-stranded RNA-specific adenosine deaminase | DNA binding protein, RNA binding protein, deaminase, kinase activator, defense/immunity protein | Chr 2: 208.5 Mb | 27.9 | 36 |
| Uso1 | USO1 vesicle transport factor | poly(A) RNA binding, protein transporter activity | Chr 14: 17.3 Mb | 27.7 | 37 |
| Sec23b | SEC23B isoform CRA_b | G-protein modulator | Chr 3: 145.2 Mb | 27.5 | 36 |
| Atg13 | Autophagy related 13 | protein kinase binding | Chr 3: 87.3 Mb | 27.4 | 37 |
| Mxd4* | Max dimerization protein 4 | DNA binding | Chr 14: 82.5 Mb | 27.2 | 33 |
| Prmt2* | Protein arginine methyltransferase 2 | androgen receptor binding, beta-catenin binding, estrogen receptor binding | Chr 20: 15.3 Mb | 27.1 | 37 |
| Arf4 | ADP-ribosylation factor 4 | epidermal growth factor receptor binding, GTP binding | Chr 16: 2.3 Mb | 27.1 | 34 |
| Gmppb | GDP-mannose pyrophosphorylase B isoform CRA_a | nucleotidyltransferase activity | Chr 8: 116.2 Mb | 27.0 | 34 |
| Fam98a | Family with sequence similarity 98, member A | poly(A) RNA binding | Chr 6: 30.9 Mb | 26.8 | 32 |
| Baz1b* | Bromodomain adjacent to zinc finger domain, 1B | Chromatin binding, histone binding, histone kinase activity | Chr 12: 26.5 Mb | 26.8 | 32 |
| Ncor2 | Nuclear receptor co-repressor 2 | Chromatin binding, glucocorticoid receptor binding, ligand-dependent nuclear receptor binding | Chr 12: 38.9 Mb | 26.6 | 33 |
| Calu | Calumenin | calcium ion binding, enzyme binding, enzyme inhibitor activity | Chr 4: 56.4 Mb | 26.1 | 31 |
| Uggt1* | UDP-glucose glycoprotein glucosyltransferase 1 | UDP-glucose:glycoprotein glucosyltransferase activity, unfolded protein binding | Chr 9: 42.3 Mb | 26.1 | 32 |
| Pde4dip* | Phosphodiesterase 4D interacting protein | enzyme binding | Chr 2: 219.3 Mb | 26.0 | 31 |
| Lgr4* | Leucine-rich repeat-containing G protein-coupled receptor 4 | transmembrane signaling receptor activity | Chr 3: 107.7 Mb | 25.9 | 28 |
| Panx1 | Pannexin-1 | gap junction channel activity, ion channel binding, protease binding | Chr 8: 13.5 Mb | 25.6 | 28 |
| Atp2c1* | ATPase secretory pathway Ca2+ transporting 1 | calcium transporting ATPase activity, calcium ion binding | Chr 8: 113.4 Mb | 25.3 | 28 |
| Btbd9 | BTB/POZ domain-containing protein 9 | adult locomotory behavior, circadian behavior, circadian sleep/wake cycle, non-REM sleep | Chr 20: 11.4 Mb | 25.2 | 28 |
| Fndc3b* | Fibronectin type III domain containing 3B | poly(A) RNA binding | Chr 2: 132.8 Mb | 24.9 | 28 |
| Adamtsl2* | ADAMTS-like 2 | microfibril binding | Chr 3: 11.0 Mb | 24.8 | 29 |
| Preb | Prolactin regulatory element-binding protein | DNA binding protein | Chr 6: 36.6 Mb | 24.8 | 25 |
| LOC498369 | Neighbor of Brca1 gene 1, isoform CRA_a |  | Chr 10: 89.3 Mb | 24.7 | 25 |
| Aldh1a1* | Aldehyde dehydrogenase 1 family, member A1 | aldehyde dehydrogenase activity, benzaldehyde dehydrogenase activity, identical protein binding | Chr 1: 245.5 | 24.7 | 30 |
| Flywch1* | FLYWCH-type zinc finger 1 |  | Chr 10: 12.9 Mb | 24.7 | 25 |
| Xk* | X-linked Kx blood group | cellular calcium ion homeostasis, cellular magnesium ion homeostasis, myelination | Chr X: 15.3 Mb | 24.2 | 30 |
| Sar1a | Protein Sar1a | Small GTPase | Chr 20: 33.1 Mb | 24.1 | 27 |
| Acbd3* | acyl-CoA binding domain containing 3 | protein kinase A regulatory subunit binding | Chr 13: 104.0 Mb | 23.1 | 25 |
| Creld2* | Cycsteine-rich EGF-like domains 2 | calcium ion binding | Chr 7: 129.5 Mb | 22.7 | 21 |
| Gene.3952 |  |  | Chr 7: 15.4 Mb | 21.9 | 19 |
| Gene.5065 |  |  | Chr 1: 32.5 Mb | 20.5 | 17 |
| Fmo1* | Flavin containing monooxygenase 1 | monooxygenase activity, N,N-dimethylaniline monooxygenase activity | Chr 13: 85.6 Mb | 16.9 | 7 |
| Zfp704* | Zinc finger protein 704 | enhancer sequence-specific DNA binding | Chr 2: 114.1 Mb | 15.7 | 9 |
| Slc17a3 | Solute carrier family 17, member 3 | transmembrane transporter activity for drug, efflux, and organic anions | Chr 17: 45.4 Mb | 15.6 | 4 |

**Supplementary Table 4: Genes of the lightcyan module.** Gene function was extracted from RGD. Connectivity represents the sum of the absolute values of all pairwise correlation coefficients associated with the particular gene. Connections represents the number of pairwise correlations > |0.5| that are associated with the gene of interest. Genes are marked with an asterisk (*) if none of its associated high-confidence transcripts were annotated in the Ensembl database, but at least one is similar to an annotated transcript from the rat Ensembl database, the rat RefSeq database, or the RefSeq database from other species. Abbreviations: Chromosome (Chr), Megabase (Mb)

| Gene Symbol | Gene Name | Function | Location | Connectivity | Connections |
| --- | --- | --- | --- | --- | --- |
| Ptgs1 | Prostaglandin-endoperoxide synthase 1 | lipid binding, prostaglandin-endoperoxide synthase activity | Chr 3: 20.9 Mb | 85.5 | 105 |
| Iqgap1* | IQ motif containing GTPase activating protein 1 | mitogen-activated portein kinase binding, protein complex binding, calmodulin binding | Chr 1: 143.5 Mb | 83.8 | 105 |
| Itgb2* | Integrin subunit beta 2 | cell adhesion molecule binding, protein complex binding, protein heterodimerization activity | Chr 20: 13.9 Mb | 83.8 | 103 |
| LOC691141 | Hypothetical protein LOC691141 | integral component of memrane | Chr 8: 22.6 Mb | 82.8 | 102 |
| Hck | Hemopoietic cell kinase | protein tyrosine kinase activity | Chr 3: 155.0 Mb | 81.9 | 106 |
| Unc93b1 | Unc-93 homolog B1 | protein transporter activity | Chr 1: 226.0 Mb | 81.8 | 100 |
| Msn | Moesin | actin binding, cell adhesion molecule binding, double-stranded RNA binding | Chr X: 66.1 Mb | 81.5 | 103 |
| Nckap1l | NCK associated protein 1 like (Predicted) | GTPase activator activity, protein complex binding, protein kinase activator activity | Chr 7: 142.9 Mb | 81.4 | 106 |
| Lgals3 | Galectin-3 | signaling molecule, cell adhesion molecule | Chr 15: 28.1 Mb | 80.3 | 104 |
| Upp1 | Uridine phosphorylase 1 | uridine phosphorylase activity | Chr 14: 89.1 Mb | 80.1 | 106 |
| Csf1r | Colony stimulating factor 1 receptor | cytokine binding, macrophage colony stimulating factor receptor activity, protein homodimerization | Chr 18: 55.7 Mb | 79.8 | 103 |
| Ncf4 | Ncf4 protein | guanyl-nucleotide exchange factor | Chr 7: 119.5 Mb | 79.0 | 100 |
| Ptpn18* | Protein tyrosine phosphatase, non-receptor type 18 | non-membrane spanning protein tyrosine phosphatase activity | Chr 9: 40.7 Mb | 78.3 | 103 |
| Cd97 | CD97 molecule | G-protein coupled receptor, antibacterial response protein | Chr 19: 35.8 Mb | 78.1 | 96 |
| Prex1* | Phosphatidylinositol-3,4,5-trisphosphate-dependent Rac exchange factor | phospholipid binding | Chr 3: 169.5 Mb | 77.7 | 92 |
| Capg | Macrophage-capping protein | non-motor actin binding protein, calcium-binding protein | Chr 4: 165.2 Mb | 77.6 | 99 |
| Inpp5d | Inositol polyphosphate-5-phosphatase D | inositol-4,5-bisphosphate 5-phosphatase activity, inositol-polylhosphate 5-phosphatase activity, phosphatidyl linositol trisphosphate phosphatase activity | Chr 9: 94.5 Mb | 77.5 | 92 |
| Fermt3 | Fermitin family member 3 | integrin binding | Chr 1: 229.3 Mb | 77.4 | 87 |
| Axl | Axl receptor tyrosine kinase | myosin heavy chain binding, phosphatidyl linositol 3-kinase binding, protein heterodimerization activity | Chr 1: 83.8 Mb | 77.2 | 95 |
| Lcp2 | Lymphocyte cytosolic protein 2 | signaling molecule | Chr 10: 18.9 Mb | 77.2 | 102 |
| Sdc3* | Syndecan 3 | cell adhesion, regulation of cell migration | Chr 5: 152.6 Mb | 77.0 | 90 |
| Arhgap9 | Rho GTPase activiating protein 9 | G-protein modulator, GTPase activator activity | Chr 7: 70.8 Mb | 76.9 | 96 |
| Ucp2 | Mitochondrial uncoupling protein 2 | amino acid transporter, mitochondrial carrier protein, ribosomal protein, transfer/carrier protein, calmodulin | Chr 1: 171.7 Mb | 76.9 | 90 |
| Abcg1 | ATP binding cassette subfamily G member 1 | ATP-binding cassette (ABC) transporter, ADP/ATP binding, cholesterol transporter activity | Chr 20: 12.0 Mb | 76.9 | 100 |
| Cd5l* | Cd5 molecule-like | scavenger receptor activity | Chr 2: 206.1 Mb | 75.8 | 103 |
| Laptm5 | Lysosomal multispanning membrane protein 5 | transporter | Chr 5: 152.8 Mb | 75.8 | 93 |
| Spi1 | Transcription factor PU.1 | transcription factor, signaling molecule, nucleic acid binding | Chr 3: 86.6 Mb | 75.6 | 91 |
| Mcm2 | Protein Mcm2 | DNA helicase, helicase | Chr 4: 186.1 Mb | 75.2 | 101 |
| Mob3a | MOB1, Mps One Binder kinase activator-like 2A (Yeast) | kinase activator | Chr 7: 12.0 Mb | 75.1 | 92 |
| Hcls1 | Hematopoietic cell specific Lyn substrate 1 | basic helix-loop-helix transcription factor, non-motor actin binding protein | Chr 11: 69.8 Mb | 74.9 | 95 |
| Flna | Filamin alpha | non-motor actin binding protein | Chr 1: 152.2 Mb | 74.4 | 86 |
| Stk10 | Serine/threonine kinase 10 | identical protein binding, protein homodimerization activity, protein serine/threonine kinase activity | Chr 10: 17.4 Mb | 74.2 | 84 |
| Emr1 | EGF-like module-containing mucin-like hormone receptor-like 1 | G-protein coupled receptor, antibacterial response protein | Chr 9: 8.5 Mb | 74.1 | 96 |
| Ncoa3* | Nuclear receptor coactivator 3 | androgen receptor binding, estrogen receptor binding, ligand-dependent nuclear receptor transcription coactivator activity | Chr 3: 168.9 Mb | 74.0 | 87 |
| Cyth4* | cytohesin 4 | ARF guanyl-nucleotide exchange factor activity | Chr 7: 119.8 Mb | 74.0 | 92 |
| Ptafr | Platelet-activating factor receptor | G-protein coupled receptor | Chr 5: 154.4 Mb | 73.9 | 99 |
| Gnai2 | Guanine nucleotide-binding protein G(i) subunit alpha-2 | heterotrimeric G-protein | Chr 8: 115.7 Mb | 73.9 | 98 |
| Fcer1g | Fc fragment of IgE receptor Ig | IgE binding and receptor activity, IgG binding | Chr 13: 94.2 Mb | 73.7 | 102 |
| Rac2 | Protein Rac2 | small GTPase | Chr 7: 119.8 Mb | 73.5 | 87 |
| Cotl1* | Coactosin-like F-actin binding protein 1 | actin binding | Chr 19: 63.2 Mb | 73.5 | 85 |
| Myo1f | Myosin IF (Predicted) | G-protein modulator, actin binding motor protein, cell junction protein | Chr 7: 18.7 Mb | 73.1 | 91 |
| Aif1 | Allograft inflammatory factor 1 | annexin, calmodulin | Chr 20: 7.2 Mb | 72.9 | 102 |
| Nfam1* | NFAT activating protein with ITAM motif 1 | transmembrane signaling receptor activity | Chr 7: 123.8 Mb | 72.8 | 92 |
| Gbp2 | Guanylate binding protein 2 | GTP binding, GTPase activity | Chr 2: 266.8 Mb | 72.7 | 93 |
| Nrros | Negative regulator of reactive oxygen species | receptor, extracellular matrix protein | Chr 11: 75.0 Mb | 72.3 | 93 |
| Hk3 | Hexokinase-3 | enzyme binding, hexokinase activity, hormone binding | Chr 17: 12.3 Mb | 71.9 | 98 |
| Anxa3 | Annexin A3 | calcium-dependent phospholipid binding, phospholipase A2 inhibitor activity, calcium dependent binding | Chr 14: 14.4 Mb | 71.9 | 90 |
| Cd38 | ADP-ribosyl cyclase 1 | cyclase, glycosidase | Chr 14: 71.8 Mb | 71.6 | 90 |
| Gpnmb | Transmembrane glycoprotein NMB | membrane-bound signaling molecule, cell adhesion molecule | Chr 4: 143.4 Mb | 71.3 | 95 |
| Gene.13348 |  |  | Chr 16: 20.4 Mb | 71.1 | 91 |
| Grn | Granulin | poly(A) RNA binding | Chr 10: 90.2 Mb | 71.0 | 85 |
| Abi3 | ABI family, member 3 | G-protein modulator | Chr 10: 83.4 Mb | 70.7 | 82 |
| Arhgap30 | Rho GTPase activiating protein 30 | Signal transduction | Chr 13: 94.4 Mb | 70.5 | 82 |
| Icam2 | Intercellular adhesion molecule 2 | signaling molecule, immunoglobulin superfamily cell adhesion molecule | Chr 10: 94.3 Mb | 70.3 | 78 |
| Npc2 | NPC intracellular cholesterol transporter 2 | cholesterol binding, enzyme binding | Chr 6: 117.1 Mb | 70.2 | 90 |
| Cfp | Complement factor properdin |  | Chr X: 2.1 Mb | 70.0 | 84 |
| Irf8* | Interferon regulatory factor 8 | DNA binding | Chr 19: 65.0 Mb | 70.0 | 80 |
| Clic1 | Chloride intracellular channel protein 1 | transferase, signaling molecule, reductase, cytoskeletal protein, epimerase/racemase, translation elongation factor | Chr 20: 7.1 Mb | 70.0 | 84 |
| Akr1b1 | Aldose reductase | reductase | Chr 4: 61.4 Mb | 69.9 | 85 |
| LOC681069* | Similar to paired immunoglobin-like type 2 receptor beta |  | Chr 12: 22.7 Mb | 69.8 | 92 |
| Plau | Plasminogen activator, urokinase receptor | urokinase plasminogen activator receptor activity, enzyme binding, protein domain specific binding | Chr 15: 3.6 Mb | 69.8 | 88 |
| Sirpa* | Signal-regulatory protein alpha | protein phosphorylated amino acid binding | Chr 3: 127.9 Mb | 69.5 | 80 |
| MGC112715 | Platelet receptor Gi24 | immunoglobulin superfamily cell adhesion molecule | Chr 20: 31.7 Mb | 69.2 | 82 |
| Tmem106a | Transmembrane protein 106A |  | Chr 10: 89.3 Mb | 68.8 | 85 |
| Cybb | CytoChrome b-245, beta polypeptide, isoform CRA_a | oxidase | Chr X: 15.4 Mb | 68.3 | 76 |
| Marco | Protein Marco | transporter, surfactant, receptor, extracellular matrix structural protein, antibacterial response protein | Chr 16: 84.6 Mb | 68.3 | 80 |
| C1qc | Complement component 1, q subcomponent, C chain | Negative regulation of granulocyte differentation | Chr 5: 159.0 Mb | 68.3 | 80 |
| Sh3bgrl3 | SH3 domain binding glutamate-rich protein like 3 | GTPase activator activity, semaphorin receptor binding | Chr 5: 156.0 Mb | 67.6 | 74 |
| Timd4 | T-cell immunoglobulin and mucin domain containing 4 | phosphatidylserine binding | Chr 10: 31.7 Mb | 67.6 | 86 |
| Rcan3* | RCAN family member 3 | troponin l binding | Chr 5: 157.5 Mb | 67.6 | 81 |
| Ripk3* | Receptor-interacting serine-threonine kinase 3 | identical protein binding, NF-kappaB-inducing kinase activity, protein complex binding | Chr 15: 38.4 Mb | 67.3 | 77 |
| Ly86 | Lymphocyte antigen 86 | positive regulation of lipopolysaccharide-mediated signaling pathway | Chr 17: 30.0 Mb | 67.2 | 75 |
| Tspan13 | Tetraspanin-13 | membrane-bound signaling molecule, receptor, cell adhesion molecule | Chr 6: 65.1 Mb | 67.0 | 87 |
| Slc15a3 | Solute carrier family 15 member 3 | proton-dependent oligopeptide secondary active transmembrane transporter activity, symporter activity | Chr 1: 234.0 Mb | 66.9 | 87 |
| Itgam | Integrin subunit alpha M | proteinheterodimerization activitym glycoprotein binding, heparan sulfate proteoglycan binding | Chr 1: 206.5 Mb | 66.5 | 79 |
| Pycard | PYD and CARD domain containing | cysteine protease, protease inhibitor | Chr 1: 206.5 Mb | 66.2 | 79 |
| Lcp1 | Lymphocyte cytosolic protein 1 | non-motor actin binding protein | Chr 15: 61.0 Mb | 66.0 | 75 |
| Coro1a | Coronin-1A | non-motor actin binding protein | Chr 1: 205.1 Mb | 65.9 | 70 |
| Fgl2 | Fibrinogen-like 2 | signaling molecule | Chr 4: 10.3 Mb | 65.6 | 73 |
| C1qb | Complement component 1, q subcomponent, B chain | Homodimerization activity | Chr 5: 159.0 Mb | 65.3 | 76 |
| Ccl6 | C-C motif chemokine 6 | chemokine | Chr 10: 70.4 Mb | 64.5 | 72 |
| Ccl5 | C-C motif chemokine 5 | chemokine | Chr 10: 70.4 Mb | 63.9 | 81 |
| Pltp | Phospholipid transfer protein | lipid binding | Chr 3: 167.5 Mb | 63.4 | 73 |
| Tyrobp | TYRO protein tyrosine kinase-binding protein | identical protein binding, receptor binding | Chr 1: 90.0 Mb | 63.4 | 70 |
| Rab31 | Ras-related protein Rab-31 | GDP/GTP binding | Chr 9: 112.9 Mb | 62.9 | 70 |
| Ctss | Cathepsin S | cysteine-type peptidase activity, collagen binding | Chr 2: 217.1 Mb | 62.7 | 66 |
| Nlrc5* | NLR family, CARD domain containing 5 | RNA polymerase II core promoter sequence-specific DNA binding | Chr 19: 10.9 Mb | 62.5 | 72 |
| Gene.15155 |  |  | Chr 7: 12.9 Mb | 62.4 | 68 |
| Epsti1 | Epithelial-stromal interaction protein 1 |  | Chr 15: 63.8 Mb | 62.3 | 70 |
| Cmtm3 | CKLF-like MARVEL transmembrane domain containing 3 | positive regulation of B cell receptor signalling pathway | Chr 19: 829.2 Kb | 62.0 | 64 |
| Fcgr3a | Fc fragment of IgG, low affinity IIIa, receptor | IgG binding | Chr 13: 94.0 Mb | 61.9 | 64 |
| Vim | Vimentin | kinase binding, protein phosphatase 2A binding | Chr 17: 82.5 Mb | 61.8 | 64 |
| Csf1* | Colony stimulating factor 1 | protein homodimerization activity, cytokine activity, growth factor activity | Chr 2: 230 Mb | 61.0 | 70 |
| Gfra1 | GDNF family receptor alpha 1 | integrin binding, receptor activity | Chr 1: 286.8 Mb | 60.9 | 63 |
| Fam105a | Family with dequence similarity 105, member A |  | Chr 2: 100.1 Mb | 58.5 | 59 |
| Nkg7 | Natural killer cell group 7 sequence, isoform CRA_a | transporter, cytoskeletal protein | Chr 1: 99.6 Mb | 58.2 | 52 |
| Psmb10 | Proteasome subunit beta type-10 | threonine-type endopeptidase activity | Chr 19: 48.8 Mb | 58.0 | 59 |
| Prf1 | Perforin 1 | Calcium ion binding, wide pore channel activity | Chr 20: 32.7 Mb | 57.0 | 47 |
| Clec4f | C-type lectin domain family 4 member F | immunoglobulin receptor superfamily, cell adhesion molecule | Chr 4: 180.0 Mb | 56.3 | 47 |
| Napsa | Napsin | aspartic protease | Chr 1: 101.7 Mb | 55.6 | 57 |
| Wipf1* | WAS/WASL interacting protein family, member 1 | actin binding | Chr 3: 66.6 Mb | 54.9 | 44 |
| Itgb3* | Integrin subunit beta 3 | fibrinogen binding, peptide binding, C-X3-C chemokine binding | Chr 10: 92.4 Mb | 54.9 | 50 |
| C1qa | Complement component 1, q subcomponent, A chain | response to iron ion, complement activation, classical pathway | Chr 5: 159.0 Mb | 54.3 | 54 |
| Abca8 | ATP binding cassette subfamily A member 8 | ATP-binding cassette (ABC) transporter, ATPase activity | Chr 10: 98.0 Mb | 52.6 | 40 |
| Il1b | Interleukin 1 beta | cytokine activity, protein domain specific binding | Chr 3: 128.1 Mb | 50.5 | 36 |
| Timp1 | Metalloproteinase inhibitor 1 | metalloprotease inhibitor | Chr X: 2.2 Mb | 49.4 | 30 |
| Siva1* | SIVA 1, apoptosis-inducing factor | CD27 receptor binding, tumor necrosis factor receptor binding, virus receptor activity | Chr 6: 146.2 Mb | 48.6 | 25 |
| Aftph | Aftiphilin | clathrin binding | Chr 14: 104.7 Mb | 48.1 | 27 |
| Pla2g2d | Phospholipase A2, group IID | phospholipase | Chr 5: 161.0 Mb | 47.6 | 26 |
| LOC100911874 | Solute carrier family 40 member 1-like | iron ion transmembrane transporter activity | Chr 9: 52.6 Mb | 47.1 | 29 |
| St8sia1* | ST8 alpha-N-acetyl-neuraminide alpha-2,3-sialyltransferase 1 | sialyltransferase activity | Chr 4: 241.3 Mb | 45.3 | 15 |
| Slc11a1 | Solute carrier family 11 member 1 | manganese ion transmembrane transporter activity, metal ion proton antiporter activity, protein homodimerization activity | Chr 9: 81.4 Mb | 45.1 | 22 |
| Rpn1* | Ribophorin l | poly(A) RNA binding | Chr 4: 185.2 Mb | 44.9 | 23 |
| Mtmr7 | Myotubularin related protein 7 (Predicted), isoform CRA_a | phosphatase | Chr 16: 54.5 Mb | 44.0 | 18 |
| Ednra | Endothelin-1 receptor | G-protein coupled receptor | Chr 19: 44.8 Mb | 42.9 | 16 |
| Pex2 | Peroxisomal biogenesis factor 2 | zinc ion binding | Chr 2: 118.0 Mb | 42.1 | 21 |
| Gene.14524 |  |  | Chr 13: 101.5 Mb | 41.1 | 19 |
| AABR06046654.1 | Uncharacterized Protein |  | Chr 6: 152.0 Mb | 37.1 | 8 |
| Zkscan5 | Zinc finger with KRAB and SCAN domains 5 | metal ion binding, nucleic acid binding, transcription factor activity, sequence-specific DNA binding | Chr 12: 13.2 Mb | 34.5 | 6 |

**Supplementary Figure S1: Comparison of co-localization and shared biological pathway**. a) Co-localization occurs when multiple genes are associated with the same SNP or multiple SNPs within linkage disequilibrium, and are consequently expressed in a similar manner. b) Two genes likely share a biological pathway when one gene directly impacts the expression of another and although both are associated with the same SNP, their association remains after accounting for the variance induced by the shared SNP.

**Supplementary Figure S2: Comparison of Ensembl annotated and unannotated transcripts in the BN-Lx and SHR transcriptomes**. Based on these images, we defined that a high-confidence transcript must have an FPKM > 1. a) The total number of BN-Lx transcripts over FPKM thresholds. b) A total number of SHR transcripts over FPKM thresholds. The x-axis in both images represents the FPKM threshold used to determine the number of transcripts expressed (y-axis). For example, the first set of bars are the number of transcripts with an FPKM value greater than zero.

**Supplemental Figure S3: Cell-type specific expression of gene products in the firebrick4 module.** Cell-type Acronyms: HC represents hepatocyte cells, HSC represents hepatic stellate cells, SEC represents sinusoidal endothelial cells, and KC represents Kupffer cells. Strain Acronyms: BN-Lx represents Brown Norway with polydactyl luxate mutation, SHR represents spontaneous hypertensive rat. The color and intensity of each cell represents its relative expression compared to the strain average in the Kupffer cells, e.g. a value of 2 indicates that the expression in that strain and cell-type is twice the average expression in the Kupffer cells. Genes are in order of connectivity, with the highest connectivity at the top of the graphic and the lowest connectivity at the bottom.

**Supplemental Figure 4: Cell-type specific expression of gene products in the lightcyan module.** Cell-type Acronyms: HC represents hepatocyte cells, HSC represents hepatic stellate cells, SEC represents sinusoidal endothelial cells, and KC represents Kupffer cells. Strain Acronyms: BN-Lx represents Brown Norway with polydactyl luxate mutation, SHR represents spontaneous hypertensive rat. The color and intensity of each cell represents its relative expression compared to the strain average in the Kupffer cells, e.g. a value of 2 indicates that the expression in that strain and cell-type is twice the average expression in the Kupffer cells. Genes are in order of connectivity, with the highest connectivity at the top of the graphic and the lowest connectivity at the bottom.
